# Multiconfigurational Study on the Contribution of the Nondynamical and Dynamical Correlation Energies to the Dissociation Energies of Li_2_-to-F_2_ Molecules

**DOI:** 10.1021/acsomega.5c00734

**Published:** 2025-04-28

**Authors:** Berkay Sütay

**Affiliations:** Department of Chemistry, Istanbul Technical University, 34469 Istanbul, Turkey

## Abstract

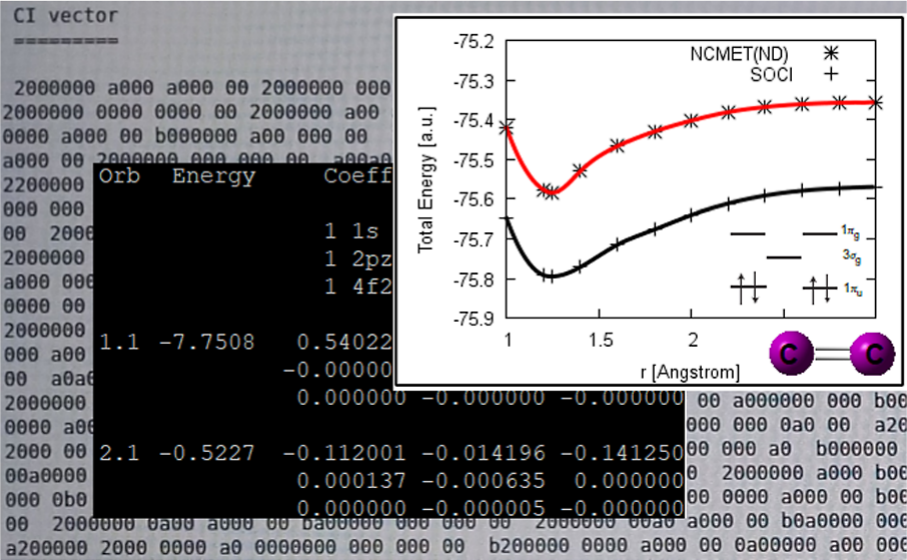

Approaching the exact solution of nonrelativistic electronic wave equation in molecules and the calculation of thermochemical quantities with high accuracy, without the help of extrapolation techniques or complicating *r*_12_ terms, is still a challenging task in quantum chemistry. Recent advances in computer hardware made it possible to achieve very high accuracy. However, it is still difficult to describe many chemically important situations where the key problem is the inherent multideterminental nature of the wave function. The inclusion of nondynamical correlation in terms of internal correlation, semi-internal correlation, and orbital polarization effects is crucial in multireference systems. The multireference character of the system at large separations, including the dissociation region, is known to be the major difficulty for the state-of-the-art theoretical calculations. Accurate estimation of PECs is contingent upon the proper treatment of the intricate interplay of nondynamical and dynamical correlation over the dissociation path. Despite their simple bonding schemes, the homonuclear diatomic molecules of the first row atoms are still notoriously difficult to describe from first-principles due to their varied electronic structures. In this work, the contribution of nondynamical and dynamical correlations to dissociation energies of diatomic molecules of first row atoms (Li_2_ to F_2_) was investigated in great detail. Starting from a large active space, the multiconfigurational type NCMET(ND) wave function was calculated which includes manually chosen nondynamical correlation terms. The def2-QZVP and correlation-consistent quadruple-ζ basis sets (aug-cc-pVQZ) have been opted in all calculations. A complete basis set (CBS) limit has also been calculated through 5Z and 6Z basis sets. Internal and semi-internal type triple, quadruple, and higher excitations were also included. The contributions of scalar relativistic effects along with the spin–orbit interaction to dissociation energy were taken into account. In addition, CASCI, CASVB, SOCI, Mk-MRCC, and NCMET (nonclosed shell many-electron theory) type calculations were performed to cover the effects of dynamical correlation. The core correlation effects including the core polarization phenomena and the corrections beyond adiabatic approximation were also considered. The results were also compared to full CI computations which are also performed in this work. The expectation values of various one-electron properties were also tested for NCMET(ND) and SOCI methods.

## Introduction

In quantum chemistry, the fundamental problem is to solve the Schrödinger equation. Exact methods for solving this equation rely on an explicit construction of the Hilbert space of the given system, in which the number of determinants required to reach a given level of accuracy will scale exponentially with the number of atoms. The main purpose of this vast body of work is to reach chemical accuracy, however, through a combination of fast computers and efficient algorithms; as of today, the prospects for a direct solution are realistic only for the lightest atoms and a few diatomic molecules. But, of course, we must apply approximate methods for larger systems of practical interest with a high level of accuracy. And yet, accuracy should not be the only property of such a method; however, robustness and also being simple enough to enable “physical insight” are also important aspects of an approximate method. The Schrödinger equation for stationary and excited states can be approximated by a matrix-eigenvalue equation
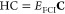
1where **H** is the representation of Hamiltonian operator in terms of configuration state functions (CSF) expressed as a linear combination of determinantal functions or N-electron symmetry eigenfunctions constructed from an orbital basis. Such orbital basis set methods provide a general approach when the number of active electrons is not large. After selection of a suitable orbital set made up of occupied (HF, Brueckner, etc.) and suitable correlation orbitals (natural or localized orbitals), the conventional configuration interaction (CI) calculation in the large N-electron space is called full CI (FCI). In a given basis, the corresponding *H*_μν_ matrix elements require hundreds of computer processor cycles and disk space. Thus, in a CSF framework, the upper limit size of the computational process is determined by the capacity of the disks employed. In the sense of FCI, N-electron wave function is written as
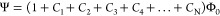
2where *C*_*n*_ (*n*: 1,2, ..., *N*) is a linear combination of all possible operators creating *n*-tuple excitations out of the HF determinant Φ_0_. Then the energy is found as
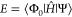
3
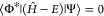
4where Φ* runs over all excited determinants. When the orbital basis set is finite, even for the systems with a few electrons, the number of variables in the system of algebraic equations becomes enormous, and these equations cannot be handled in practice. Traditional FCI is an impossible task, except the ones with too small orbital bases, and needs to be drastically simplified, resulting in several approximate methods. The simplest method of this type is CISD which limits *C*_*n*_ to single to and double excitations only. It is well-known that such an approximation becomes increasingly poorer with growing *N*. This is due to the fact that the approximate wave function is not size-extensive and the approximate energy is not size-consistent. Addition of *C*_3_ and *C*_4_ does not help and, in general, there is no easy way of simplifying eqs 3 and 4 without losing the size-extensivity and size-consistency properties in a variational calculation, which is because the complete cancellation of the denominator is only possible when the all possible excitations (all terms in full CI) are included in the nominator. A general, formally appealing, and computationally practical solution to this problem is given by the perturbational variant of many-electron theory (MET) of Sinanoğlu^1,2^ in its full form, which is known as the coupled cluster (CC) method.^3^ In the February of 1960, Lipkin pointed out the fundamental paradox of the many-body problem as “people who do not know how to solve the three-body problem are trying to solve the n-body problem”.^4^ In the October of the same year, Sinanoğlu published his work on the three-electron problem, i.e., the lithium atom, where he thoroughly presented the core polarization phenomena along with the pairwise and nonpairwise additive terms and three and more electron effects.^5^ By the use of sophisticated operator techniques and Green functions, he then showed the first-order wave function is decomposed into pair functions.^6^ In the middle of 1961, he generalized his ideas on the pair functions to a cluster ansatz on the basis of the variational principle.^1^ In MET, the wave function is written as
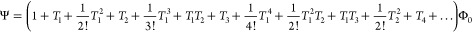
5where *T*_*n*_s are the cluster operators then, of course, *C*_*n*_s and *T*_*n*_s are inter-related in this way. This is the very well-known cluster expansion ansatz first introduced into quantum chemistry by Sinanoǧlu in 1961 (see also (7 and 8)). The coefficients of higher than double correlations (T, Q, 5, 6, etc.) are not independent parameters but are closely related to the coefficients of doubles (let it be “*c*”) as MET dictated. No matter how small the *c*^2^ is, i.e., the contribution to electronic density, if the number of electrons is large enough, then the higher correlation terms contribute significantly. He showed that the coefficients of *n*-tuply excited determinants in CI expansion can be obtained from the coefficients of determinants with lower excitation order. The theory is computationally too much demanding to be applied in its full form, e.g., the accurate solution of the first-order pair equation, also known as *Sinanoğlu equation*, tried to be solved not only numerically in the presence of 4-electron integrals but also by using Gaussian geminals^9^ where he imposed the well-known strong orthogonality (SO) condition on the pair functions and also proposed the use of *r*_12_-dependent functions which later gave birth to weak orthogonality functional, R12 methods, and RI approximation. A few approximate versions of MET have also been proposed by Sinanoǧlu’s himself in 60s, e.g., the wave function including only the powers of T_2_ cluster operator (D, Q, 6, 8, etc., excitations), say approximate MET v.1 and a more approximate one with D excitations only, say approximate MET v.2. (almost identical to variational version of its successor, the decoupled L-CPMET). The influence of triple and higher (connected quadruples, connected pentuples, etc.) correlations is taken into account inside the remainder term in the related variational energy expression of MET v.1—which is strictly an upper bound to the energy—while it is neglected in MET v.2 to save some computational time. In 1966, the perturbational variant of MET v.1, with the direct inclusion of the remainder term from the beginning, was developed by Cizek,^3^ i.e., first as coupled pair MET (CPMET, later named as CCD), then extended CPMET, and finally the coupled cluster (CC) formalism as the perturbative variant of “full MET”. The perturbational character of CPMET also makes it easier to guarantee the size extensivity of the wave function at any truncated level over MET v.1. The first model calculation of CPMET was reported in the mid-1970s; however, the first rigorous and rather convincing CCD calculation was finally achieved in 1980 (on beryllium and neon atoms). CCSD (coupled cluster singles and doubles) and CCSDT (coupled cluster singles, doubles, and triples) equations were completed in the 1980s, more than 20 years after the introduction of MET. The CCSDTQ (coupled cluster singles, doubles, triples and quadruples) method arose in the early 90s, and the explicit inclusion of higher connected correlations into CC equations could only be possible after the 2000s. For systems dominated by a single configuration around equilibrium geometry, single reference CC methods with HF reference wave function work well. But in practice, even the CCSDT method (scales as iterative *n*^8^ where n is the number of basis functions) itself is computationally too expensive for routine work. A variety of CC methods with an approximate treatment of triples have been proposed and among these, CCSD(T) (coupled cluster singles, doubles, and noniterative triples) is useful when the fully implemented triples model (CCSDT) is expected to perform well enough.^10^ However, its agreement with experimental data arises from the fortuitous cancellation of the errors such as the approximate treatment of triples, the neglect of higher excitations, and the basis set truncation, i.e., the errors arising from the approximate or incomplete treatment of the one- and N-electron spaces.^11−14^ For instance, CCSDT predicts the dissociation energy of the dicarbon molecule 2 kcal mol^–1^ less than CCSD(T). However, rather accurate energies do not guarantee the accurate chemical properties, e.g., CCSD(T) may produce lower energies than high level multireference (MR) methods—the (T) term also converges much more rapidly than CCSD^15^—but the errors may still be larger because even small inconsistencies in total electronic energies may cause serious errors in energy differences of chemical interest. The multiply bonded systems require higher levels of theory, e.g., for the N_2_ molecule, even the CCSDTQ method is not enough, it diverges before reaching the 2*R*_e_ region,^16^ and the CCSDTQ56 method is required for even a qualitative description.^17^

Single reference CC and MPn methods are size-extensive but not size-consistent, and they fail at stretched geometries in the presence of strong correlation effects, which is an indication to an inherently multiconfigurational (MC) situation. Correlation effects are normally partitioned into nondynamical and dynamical correlation.^18^ Qualitatively they differ in the way they separate the electrons. Dynamical correlation is of short range, and it deals with the interaction between two electrons in the cusp region. It generally becomes less important at stretched geometries, i.e., the dissociation region, since the electrons are further apart. It is local and therefore more important for small separations and generally added to MCCI wave functions, etc., e.g., by perturbation theory. The cancellation of much of it in dissociation energy is also well-known. Nondynamical correlation is of long range and ensures a correct balance between ionic and covalent components of the wave function. It is a Coulomb correlation that permits electrons to avoid one another leading to a large separation in the space of a pair of electrons by reducing their mutual repulsion with respect to a zeroth-order wave function, i.e., the HF wave function. The orbitals used to take into account the effects of nondynamical correlation define the *active space* which is the set of orbitals whose occupations vary among the different configurations included in a multiconfigurational configuration interaction (MCCI) wave function. A FCI wave function defined in the valence active space is then called valence CASCI (valCASCI) wave function. The difference between HF and valCASCI energies is called the internal correlation energy. The valCASCI recovers the whole internal correlation; however, it completely lacks semi-internal, polarization, and external (dynamical) correlations.^19^ Then, the difference between the valCASCI and FCI energies is the sum of those remaining correlations. Internal correlations are described by the configurations built entirely from the valence active space. This kind of correlation also includes near-degeneracy effects. Semi-internal correlation is described by the configurations built from the orbitals of the valence active space plus one correlation orbital. In terms of the formulation of nonclosed shell many-electron theory (NCMET),^19^ this kind of correlation is coupled with orbital average spin and symmetry polarization effects which are simply the nondynamical type single excitations^18^ required to correct the SCF solution and different in nature from dynamical type single excitations, the effect of which is simply to rotate the orbitals. External (dynamical) correlation is described by configurations in which two or more orbitals are outside the valence active space. In closed shell systems (He, Ne, He_2_, Ar_2_, etc.), the entire correlation is of external type correlations.

Quantitatively, for many chemical applications, the molecular wave function is also important, since observables other than the energy are often required. In the treatment of nonclosed (open) shell systems, spin-contaminated or symmetry-broken wave functions are inappropriate in such cases. Spin-dependent properties are also determined wrong using an unrestricted HF reference. The explicit calculation of nondynamical correlation and the related spin and symmetry orbital average polarization effects in nonclosed shell systems require the use of multireference methods. The first multireference theory for nonclosed shell systems is NCMET^19^ which was proposed in 1964 as the nonclosed shell version of MET, again as a variational theory. It starts with a reference space including the nondynamical correlations (internal corr. + semi-internal corr. + polarization effects) and then takes into account the dynamical (external) correlations in terms of cluster expansion ansatz. In this sense, NCMET may be considered as the central theory, and MET is its closed shell version which only includes external correlations. NCMET is variational and size-consistent but is hard to perform in its full form. NCMET(ND) is its truncated version, which only includes the nondynamical correlation part. It covers the effects of higher excitations such as the internal triples, quadruples, pentuples, hextuples, etc., and semi-internal triples, quadruples, etc. (then the rest of the triple, quadruple, etc., excitations are of external type correlations and not a part of NCMET(ND) wave function). These high level correlations enter quite soon into the NCMET(ND) wave function. As an MCCI type expansion, NCMET(ND) is the most compact wave function taking care of whole nondynamical correlation and may be a well-designed starting point for quantum Monte Carlo (QMC) calculations to consider the dynamical correlation effects. In the present work, such an effort will be performed via near-NCMET which is an approximation to “full NCMET”. It is also possible to form a compact wave function including external correlation as well. First-order interacting space (FOIS) is one such approach^20^ along with the second-order configuration interaction (SOCI) method—slightly larger space than FOIS-where the reference function is chosen as valCASCI wave function. SOCI (or equivalently the uncontracted MR(valCAS)CISD) may be considered as an approximation to full NCMET. Alternatively, multireference configuration interaction (MRCI) and multireference coupled cluster (MRCC) methods try to overcome these difficulties by explicitly modeling both dynamical and nondynamical correlation. In the MRCI method, the choice of the references and truncation of single and double excitation space must be performed carefully so as not to bias the results. One possibility is to select an *a priori* subspace, e.g., NCMET(ND) or SOCI, having invariant properties under the unitary transformations of occupied orbitals and the sets of correlation orbitals, giving rise to MRCI, complete active space configuration interaction (CASCI), and restricted active space configuration interaction (RASCI). Configuration interaction using a perturbative selection made iteratively (CIPSI)^21^ is also a road to have larger compact wave functions. Although MRCI and MRCC methods explicitly include the multireference character of the nonclosed shell system, the choice of the active space does not depend on a particular strategy.

Despite their simple bonding schemes, the homonuclear diatomic molecules of the first row atoms are still notoriously difficult to describe from first-principles due to their varied electronic structures. The goal of this study is to discuss the contribution of nondynamical and dynamical correlation to dissociation energy and their role in dissociation phenomena. The importance of a balanced description of nondynamical correlation effects for the modeling of multireference systems supports the strategy behind this work. Besides its systematic nature and computational efficiency, this model enables us to interpret the results of electronic structure calculations performed in large basis sets using a simple molecular orbital picture in a valence space of bonding and antibonding orbitals. A subsequent application of MRCI-SD on top of the NCMET(ND) wave function in the QZ level of basis at least, would appear to be a felicitous combination for a compact and balanced way to produce near-NCMET results which both include nondynamical and dynamical correlations. The NCMET(ND) wave function is such a trial function, handling a limited number of determinants that contribute the most to the total energy in each type of excitation (S, D, T, Q, 5, 6, etc.) instead of a formidable number of determinants as in CISDTQ, CISDTQ5, etc.

## Computational Details

Multiconfigurational NCMET(ND) wave function, as a reference, in the present work is always a function in the space of a small number of CSFs that are generated in *D*_2*h*_ abelian point group symmetry by the number of valence electrons occupying a relatively small number of MOs. NCMET(ND) calculations were performed in MCCI formalism, i.e., RASCI, by selecting all internal, semi-internal, and polarization terms manually with respect to the spin and spatial symmetry restrictions for each atom and diatomic molecule. The active space was chosen to be of sufficient size for the convergence of the semi-internal correlation (see Supporting Information). The core electrons are kept frozen in all calculations, unless otherwise stated. The effects of core correlation (core–core correlation and core polarization) and relativistic corrections along with the spin–orbit (SO) interaction were calculated and eliminated from the experimental dissociation energies for a reasonable comparison to nonrelativistic infinite mass energies. Full atomic symmetry is imposed in calculations on the atomic asymptotes for unrestricted systems which are based on ROHF wave functions, i.e., the symmetry-equivalent atomic asymptotes are chosen to be described by symmetry-equivalent orbitals. Reliable results comparable to experimental data can be obtained when a basis set is used that is at least large enough to include f functions.^22^ The def2-QZVP basis set of Weigend and Ahlrichs^23^ was used in NCMET(ND) computations. The augmented versions of the correlation-consistent polarized valence and polarized core and valence basis sets of Dunning and co-workers,^24−27^ denoted by aug-cc-pVXZ and aug-cc-pCVXZ, up to *X* = 6 hextuple zeta, were also used which are constructed by adding shells of s, p, d, f, g, etc., type correlating functions to the contracted HF orbitals and might be expected to converge systematically to the CBS limit. The polarization functions are defined by spherical harmonics. The orbitals for the SOCI calculations were taken from valCASCI calculations. The SOCI calculations were carried out by using the internally contracted MRCI code of Werner and Knowles.^28,29^ SOCI actually needs to be performed under uncontracted MRCI; it was found that the differences between uncontracted and internally contracted MRCI for dissociation energies are lower than 0.5 kcal mol^–1^.^30^ At this juncture, it is also worth stressing that the cluster corrections are not applied in SOCI energies because the SOCI calculations in full valence active space were found more reliable without such corrections, i.e., +*Q* correction was found to result in double counting some effects.^31^

Quite akin to those ab initio methods relying on the wave functions expanded in determinantal form, NCMET and SOCI methods too suffer from the poor convergence toward the CBS limit. The computed energies are extrapolated to the CBS limit in an attempt to eliminate the basis set truncation error. The complete basis set extrapolations were performed by using exponential extrapolation for the HF part and the extrapolation with respect to maximum angular momentum in the basis^32^ for the correlation part. Dissociation energies predicted in the present work were calculated by the supermolecular approach to overcome the size-consistency problem, i.e., the separated atom limit is replaced by a highly stretched geometry, i.e., 30 Å for all molecules (except for Be_2_ at 50 Å). In order to gain a more quantitative insight into the quality of this wave function, the nonparallelity error (NPE) of the PECs along with the size-consistency error (SCE) was also calculated.

When a well-ordered set of MOs is chosen, it is known that higher excitations, e.g., T, Q, 5, 6, etc., into higher lying virtual space make negligible contributions compared to the same-order excitations into the lower virtual space. NCMET(ND) and SOCI work on this basis. Connected and disconnected Q, 5, 6, 7, and 8 electron correlation terms are of paramount importance in determining both the dissociation energy and the related spectroscopic parameters, e.g., the effect of connected pentuples, especially for multiple bonds, is found prominent in various studies.^33−35^ All the possible “nondynamical type” higher-order correlation terms beyond connected quadruples were included in NCMET(ND) wave function, e.g., up to quadruples for Be_2_ (4 valence electrons), hextuples for B_2_ (6 valence electrons), octuples for C_2_ (8 valence electrons), etc., to support the correct asymptotic convergence of the PEC (the NCMET(ND) means the inclusion of all types of available excitations included throughout in this work). Specially, semi-internal triple^36−38^ and quadruple excitations play an essential role in this sense which are directly included in NCMET(ND) expansion. Dynamical parts of these high-order correlations were included in SOCI and near-full NCMET wave functions.

To facilitate a reasonable comparison between theory and experiment, the experimental dissociation energies were modified to eliminate the SO splitting and the relativistic effects. The diagonal Born–Oppenheimer correction (DBOC) is found negligible, e.g., it contributes to the dissociation energy of homonuclear diatomics in the order of 0.01 kcal/mol.^39,40^ Core correlation along with the core polarization effects were estimated from the CCSD(T)/CBS all-electron calculations. Scalar relativistic effects were calculated as a perturbative correction using the Cowan–Griffin operator. The Breit terms and QED corrections were also neglected, e.g., for the Be_2_ molecule, these are calculated in the order of 0.013 and 0.0009 kcal mol^–1^, respectively,^41^ and even for the F_2_ molecule, they are calculated as 0.06 and 0 kcal/mol, respectively.^42^ ZPE values were calculated by using the MP2 method on a 6-31G(d) basis with a scaling factor of 0.97. The first-order SO corrections were calculated by using the CISD method. Experimental dissociation energies were taken from the most recent literature value available, i.e., for Li_2_,^43^ Be_2_,^41^ B_2_,^44^ C_2_,^45^ N_2_,^45^ O_2_,^45^ and F_2_.^45^ The estimated correlation energies were taken from ref (46) except Li_2_ and Be_2_. The correlation energy of Li_2_ was estimated from the nonrelativistic, infinite nuclear mass atomic energy^47^ and the experimental dissociation energy, after the spin–orbit and relativistic effects have been subtracted. The correlation energy of Be_2_ was explicitly estimated from full CI calculations by the author. NCMET(ND), SOCI, near-NCMET, full CI calculations, and relativistic corrections were performed in the MOLPRO^48^ package. CIPSI calculations were performed in Quantum Package^49^ program. Mukherjee’s multireference coupled cluster (Mk-MRCC),^50^ CISDTQ, and CISDTQ56 calculations were performed in PSI4 suite.^51^ All calculations were carried out on a Linux cluster that consists of machines with 512 GB memory and Intel Xeon CPUs at 2.40 GHz.

## Results and Discussion

### Atoms

The nondynamical correlation energies of the first row atoms and their partitioning into internal and semi-internal correlation types are summarized in Table 1.

**Table 1:** Frozen-Core NCMET(ND) Nondynamical Correlation Energies (au) of First Row Atoms in def2-QZVP Basis in Infinite Nuclear Mass Approximation (the Values in Parentheses Indicate the Percent Nondynamical Corr. Out of Valence Corr. Energy)

	HF energy	internal corr.	semi-internal corr. + polarization	nondynamical corr.	valence corr. energy^a^
Li	–7.4327152	0	0	0^b^	∼0
Be	–14.5730010	–0.0410	∼0^c^	–0.0410 (91.6)	–0.04478^d^
B	–24.5290184	–0.0304	–0.0235	–0.0539 (73.4)	–0.07342
C	–37.6885451	–0.0172	–0.0404	–0.0576 (56.8)	–0.10140
N	–54.4008143	0	–0.0480	–0.0480 (37.0)	–0.12951
O	–74.8092061	0	–0.0440	–0.0440 (22.5)	–0.19574
F	–99.4090549	0	–0.0261	–0.0261 (10.1)	–0.25903

a Estimated full CI valence correlation energy from ref (46) except Li and Be.

b The frozen-core NCMET(ND) is identical to ROHF for the lithium atom.

c Semi-int. corr. is found zero, and polarization contribution is found −2 × 10^–5^ au. Double excitations type dynamical corr. of Be in the valence shell is estimated in the order of −3 × 10^–3^ au.

d The correct value after the three-body effects has been added to −0.04617 au.

The contribution of S type excitations into the valence correlation of the lithium atom is almost zero. The strong internal correlation in the beryllium atom is highly prominent. The orbital average polarization effects in the valence shell of the boron atom are found to be −0.0235 au which is an improvement over the value (−0.02182 au) calculated by Schaefer and Harris^52^ and mostly arise from 2s orbitals. They also calculated 90% of valence correlation energy. In the present work, 98.5% of valence correlation has been covered in def2-QZVP basis. The portion of semi-internal correlation along with the polarization effects is calculated as −0.0235 au and found almost equal to the value −0.025 au, extracted—and estimated—from ref (53). The semi-internal correlation in the carbon atom is calculated as −0.0404 au which is consistent with the value −0.0370 au in ref (54). The nondynamical correlation is found as −0.0576 au and again consistent with the −0.059 value extracted—and estimated—from ref (54). Semi-internal triples are found significant for the carbon atom, which is calculated in the order of −0.04 millihartree. The internal correlation in the ground state nitrogen, oxygen, and fluorine atoms is zero due to symmetry restrictions. A number of all electron calculations—including QMC with Jastrow factors—were reported^55−59^ which cover more than 90% of correlation energy.

The valence, core, and total correlation energies were summarized in Table 2. Core correlation values are consistent with the literature.^63,64^

**Table 2:** Frozen-Core SOCI, Near-NCMET, and Full CI Correlation Energies (au) of First Row Atoms in def2-QZVP Basis

		valence corr.	core corr.	total corr. energy
Li	SOCI		–0.04534	–0.04534^a^
	near-NCMET			
	full CI			
Be	SOCI	–0.04568	–0.04817	–0.09434^b^^,^^c^
	near-NCMET	–0.04568		
	full CI	–0.04617^d^		
B	SOCI	–0.07198	–0.05143	–0.12485^e^
	near-NCMET	–0.07228		
	full CI	–0.07342		
C	SOCI	–0.09644	–0.05500	–0.15640^e^
	near-NCMET	–0.09860		
	full CI	–0.10140		
N	SOCI	–0.11978	–0.05880	–0.18831^e^
	near-NCMET	–0.12460		
	full CI	–0.12951		
O	SOCI	–0.18430	–0.06220	–0.25794^e^
	near-NCMET	–0.19530		
	full CI	–0.19574		
F	SOCI	–0.23191	–0.0655	–0.32453^e^
	near-NCMET	f		
	full CI	–0.25903		

a Reference (60).

b Reference (61).

c Reference (62).

d This value is reduced to −0.04478 au after three-body effects have been added.

e Reference (47).

f This energy is not accessible. It requires more than 10^19^ determinants in the QZ space.

### Homonuclear Diatomic Molecules

The nondynamical correlation energies of the homonuclear diatomics of first row atoms and their partitioning into internal and semi-internal correlation types are summarized in Table 3.

**Table 3:** Frozen-Core NCMET(ND) Nondynamical Correlation Energies (au)^a^ of the Diatomic Molecules of First Row Atoms in def2-QZVP Basis in Infinite Nuclear Mass Approximation (the Values in Parentheses Indicate the Percent Nondynamical Corr. Out of Valence Corr. Energy)

	*R*_e_ (Å)	HF energy	internal corr.	semi-internal corr. + polarization	nondynamical corr.	valence corr. energy^a^
Li_2_	2.672	–14.871461	–0.0051	–0.0148	–0.0199 (61.4)	–0.03240
Be_2_	2.4536	–29.134058	–0.0318	–0.0331	–0.0649 (60.2)	–0.10810
B_2_	1.590	–49.090857	–0.0698	–0.0565	–0.1263 (58.2)	–0.21700
C_2_	1.2478	–75.406258	–0.1285	–0.0700	–0.1985 (48.8)	–0.40686
N_2_	1.0972	–108.992420	–0.0596	–0.0630	–0.1226 (28.4)	–0.43130
O_2_	1.201	–149.667620	–0.0486	–0.0790	–0.1276 (23.8)	–0.53603
F_2_	1.4118	–198.772192	–0.0508	–0.0633	–0.1141 (18.3)	–0.62577

a Estimated full CI valence correlation energy from ref (46) except Li_2_ and Be_2_.

The valence, core, and total correlation energies were summarized in Table 4. Core correlation values are consistent with the literature.^63,64^

**Table 4:** Frozen-Core SOCI, Near-NCMET, and Full CI Correlation Energies (au) of First Row Diatomics in def2-QZVP Basis

		valence corr.	core corr.	total corr. energy^c^
Li_2_	SOCI	–0.03190	–0.09095	–0.12335
	near-NCMET	–0.03190		
	full CI	–0.03190		
	full CI^a^	–0.03240		
Be_2_	SOCI	–0.10580	–0.09670	–0.20480
	near-NCMET	–0.10580		
	full CI	–0.10582		
	full CI^a^	–0.10810		
B_2_	SOCI	–0.21315	–0.10410	–0.32110
	near-NCMET	–0.21661		
	full CI	–0.21698		
	full CI^a^	–0.21700		
C_2_	SOCI	–0.38818	–0.11230	–0.51916
	near-NCMET	–0.39630		
	CIPSI + PT2	–0.39810		
	full CI^a^	–0.40686		
N_2_	SOCI	–0.39750	–0.11903	–0.55033
	near-NCMET	–0.40770		
	CIPSI + PT2	–0.41300		
	full CI^a^	–0.43130		
O_2_	SOCI	–0.47620	–0.12488	–0.66091
	near-NCMET	–0.5071^b^		
	CIPSI + PT2	–0.50860		
	full CI^a^	–0.53603		
F_2_	SOCI	–0.54845	–0.13084	–0.75661
	near-NCMET	–0.5786^b^		
	CIPSI + PT2	–0.58710		
	full CI^a^	–0.62577		

a Estimated full CI valence correlation energy from ref (46) except Li_2_ and Be_2_.

b Could not get completed within 30 days of computation (requires 170 GB memory) and these nonconverged values correspond to the last iteration available.

c Estimated all-electron correlation energies as the sum of FCI valence correlation and core correlation.

The dissociation energies calculated at the NCMET(ND) level of theory are summarized in Table 5, which were calculated according to eq 6.
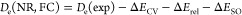
6

**Table 5:** Dissociation Energies (kcal/mol) Calculated from Different Levels of Theories in def2-QZVP Basis^a^^,^^e^

	*D*_e_ (exp)	Δcore	Δrel + ΔSO	*D*_e_ (NR,FC)	*D*_e_^b^ (NCMET(ND))	*D*_e_^c^ (SOCI)	ZPE
Li_2_	24.4	0.2	∼0	24.2	17.4 (72)	23.8 (24.1)	0.5
Be_2_	2.67	0.21	–0.01	2.47		1.63 (2.28)	0.4
B_2_	70.0^d^	0.8	–0.11	69.4	70.5 (101)	65.4 (65.9)	1.5
C_2_	147.3	1.5	–0.35	146.2	139.8 (96)	144.0 (145.6)	2.6
N_2_	228.4	0.9	–0.13	227.6	213.4 (94)	223.8 (227.2)	3.4
O_2_	120.6	0.3	–0.64	121.0	113.0 (93)	115.4 (117.3)	2.3
F_2_	38.3	–0.1	–0.80	39.2	29.4 (75)	33.7 (35.0)	1.4

a Core correlation values from Table 4. Relativistic and SO corrections from Table S1.

b The values in parentheses correspond to percent NR dissociation energy covered.

c The values in parentheses correspond to the SOCI results in aV6Z basis.

d 70 ± 14 kcal mol^–1^ [ref (44)].

e ZPE values were also reported.

The NR dissociation energies at frozen-core approximation were also shown to investigate the percent of energy covered by NCMET(ND) and SOCI levels of theories out of NR(FC) dissociation energy for each molecule. At this juncture, it is worth stressing that many different factors affecting the dissociation energy beyond nonrelativistic infinite mass energy must be considered. For this purpose, scalar relativistic effects, spin–orbit interaction, core correlation, Breit interaction, QED correction, and diagonal BO correction are all taken into account and their contributions were eliminated from the experimental dissociation energy to get the NR(FC) dissociation energy.

For C_2_ and F_2_ molecules, the percent dissociation energies covered in the presence of nondynamical correlation only are also consistent with the values reported by Braïda et al.^65^ For the O_2_ molecule where semi-internal correlation is dominant, the NCMET(ND) gives 93% of NR(FC) dissociation energy, i.e., 20–25% higher than their value which is reported as 70%. In the case of N_2_, the percent dissociation energy from NCMET(ND) is also 10% higher than their value.

The dissociation energy of the Li_2_ molecule at the valCASSCF level is found to be 23.8 kcal mol^–1^ that corresponds to 98% of the experimental value, and PEC is smooth through 30 Å. For the Be_2_ molecule, valCASSCF PEC is repulsive. The PECs for the molecules from B_2_ to F_2_ that are calculated from the valCASSCF method typically diverge, except B_2_ and N_2_, e.g., divergence starts at 2.8 Å for O_2_, 3.2 Å for F_2_, and 5 Å for C_2_. The reason for this divergence is not actually the symmetry breaking but the lack of 5, 6, 7, and 8 electron correlations due to the symmetry restrictions and also the absence of semi-internal double, triple, and higher correlations. The dissociation energy of the B_2_ molecule at the valCASSCF level is found to be 61.4 kcal mol^–1^ that corresponds to 90% of the experimental value. Likewise, that one finds 143 kcal mol^–1^ for C_2_ (98% of D_e_), 213.1 kcal mol^–1^ for N_2_ (93% of *D*_e_), 96 kcal mol^–1^ for O_2_ (80% of *D*_e_), and 19 kcal mol^–1^ for F_2_ (50% of *D*_e_). Table 6 shows the contributions of HF and correlation parts to the nonrelativistic dissociation energy. The nondynamical correlation part is also partitioned into internal and semi-internal correlation contributions as Δint and Δs-int, respectively.

**Table 6:** Dissociation Energies (kcal/mol) Calculated by NCMET(ND)/def2-QZVP in Comparison to Full CI (Exact Nonrelativistic (NR), Infinite Nuclear Mass Energy, FC) Contributions

	NCMET (ND)				full CI			
	ΔHF	Δcorr^a^	Δint	Δs-int	*D*_e_ (NR,FC)	ΔHF	Δcorr^b^	ND %^c^
Li_2_	3.8	13.6	9.2	4.4	24.2	4.6	19.6	70 (98)
Be_2_	–7.5	6.28	1.01	5.27	2.47	–7.40	9.87	64 (70)
B_2_	20.6	50.0	28.2	21.8	69.4	20.7	48.7	102 (86)
C_2_	18.3	121.5	87.8	33.7	146.2	22.2	124.0	97 (99)
N_2_	120	93.4	91.0	2.4	227.6	120.0	107.6	87 (85)
O_2_	30.9	82.1	44.0	38.1	121.0	30.0	91.0	90 (72)
F_2_	–28.8	58.2	45.8	12.4	39.2	–28.5	67.7	86 (71)

a Contribution of nondynamical type valence correlation energy.

b Contribution of full valence correlation energy.

c The values in parentheses correspond to valCASSCF contributions.

In Table 6, ΔHF contributions were predicted from HF limits and Δcorr values from the subtraction of ΔHF values from the experimental *D*_e_ energies after ΔSO and Δrel values have been extracted. In contrast to valCASSCF results, the PECs calculated from NCMET(ND) and SOCI methods predict smooth, asymptotically well-converged profiles. In spite of the multireference character and the related benchmark studies on C_2_ and N_2_ molecules, B_2_ is a pathologically multireference molecule with a large T_1_ diagnostics value and—maybe surprisingly—the F_2_ molecule seems to be the most complicated system in this series. A variety of diagnostics for the B_2_ molecule at the CCSDTQ level of theory also support our conclusion.^66^ The percent of nondynamical correlation for C_2_ and N_2_ molecules is consistent with the predictions of Dunning et al.^67,68^ The contribution of dynamical correlation to the dissociation energy of the C_2_ molecule is on the order of 3% which is directly attributed to its very strong MR character. In addition to this, the internal type connected triples and quadruples in the C_2_ molecule are found important which is strongly related to the quasi-degenerate nature of 2σ_u_ and 1π_u_ orbitals. In spite of its strong MR nature, the percent correlation covered by NCMET(ND) for the N_2_ molecule is lower than B_2_, C_2_, and O_2_ due to the presence of a triple bond. Since the N_2_ molecule has more valence electrons and shorter bond length than C_2_ and O_2_, one would expect that the electrons are more densely packed in the molecule compared to nitrogen atoms, and as a result, the contribution of dynamical correlation to dissociation energy becomes small but still larger. In the case of O_2_ and F_2_ molecules, the contribution of semi-internal correlation along with the polarization effects seems essential, which is why valCASSCF underestimates the dissociation energy of these molecules in comparison to NCMET(ND). On the other hand, it seems that valCASSCF overestimates the dissociation energy of the Li_2_ molecule while its multireference character is not as strong as B_2_, C_2_, and N_2_. The reason for this case seems to be due to the overestimation of the internal correlation of Li_2_ by the valCASSCF method. It gives −0.0316 au, i.e., more than six times of the internal correlation contribution from NCMET(ND) which is equal to −0.0051 au. However, for the rest of the molecules, valCASSCF gives 2—or less than 2—times of the internal correlation that is found by NCMET(ND), e.g., valCASSCF internal correlation energies are found to be −0.0886 au for Be_2_, −0.1302 au for B_2_, −0.2377 au for C_2_, −0.1484 au for N_2_, −0.1032 au for O_2_, and −0.0792 au for F_2_ in def2-QZVP basis.

In the long-range region of the PEC, HF contribution degrades and that is why correlation energy increases in the absolute value, Table 7. Almost for all studied molecules, the nondynamical correlation becomes twice at the long range; however, the dynamical correlation almost remains the same for C_2_, slightly increases for Li_2_ and F_2_, but changes more significantly for B_2_, N_2_, and O_2_. In terms of NCMET(ND) energies at 30 Å separation, the total electronic energy of the Li_2_ molecule approaches the true dissociation limit with 2 times the atomic ROHF energies as expected. The corresponding size-consistency error is only 0.8 kcal mol^–1^. The NCMET(ND) minimum of the Be_2_ molecule is found at almost 4.5 Å (see the later discussion about that). The NCMET(ND) energies of B_2_, C_2_, and N_2_ molecules at 30 Å separation approach 2 times those of atomic NCMET(ND) energies with large SCE values due to the strong internal and semi-internal correlations in their atomic counterparts. The NCMET(ND) energies of O_2_ and F_2_ molecules at 30 Å separation approach 2 times those of atomic ROHF energies with small SCE values (1.9 and 0.7 kcal mol^–1^, respectively) due to the lack of internal correlation in oxygen and fluorine atoms.

**Table 7:** Correlation Energies (au) at Equilibrium and Dissociation Regions

		ND	D	sum
Li_2_	*R*_e_	–0.0199	–0.0125	–0.0324
	∞	–0.0419	–0.0221	–0.0640
Be_2_	*R*_e_	–0.0649	–0.0432	–0.1081
	∞	–0.0749	–0.0489	–0.1238
B_2_	*R*_e_	–0.1263	–0.0907	–0.2170
	∞	–0.2060	–0.0888	–0.2948
C_2_	*R*_e_	–0.1985	–0.2084	–0.4069
	∞	–0.3921	–0.2132	–0.6053
N_2_	*R*_e_	–0.1226	–0.3087	–0.4313
	∞	–0.2715	–0.3313	–0.6028
O_2_	*R*_e_	–0.1276	–0.4084	–0.5360
	∞	–0.2585	–0.4226	–0.6811
F_2_	*R*_e_	–0.1141	–0.5117	–0.6258
	∞	–0.2069	–0.5269	–0.7338

It should also be noted that the NCMET(ND) method predicts the dissociation energy of the B_2_ molecule which is significantly below the atomic limit but still within the ±14 kcal/mol error bar of experimental value, i.e., two boron atoms, and one can argue that this is due to the essence of dynamical correlation. In contrast to the other systems in the Li_2_–F_2_ sequence of diatomics, B_2_ is the second system after Be_2_ for which the dynamical correlation is somewhat essential but still not as strong as in the case of Be_2_. A supporting clue for this idea also seems to be the decrease of the NPE value for the B_2_ molecule from 7 kcal mol^–1^ (for NCMET(ND)) to 0.4 kcal mol^–1^ (for SOCI). The largest bond length of B_2_ (1.6 Å) in B_2_–F_2_ series is also responsible for less densely packed electrons which reveals the importance of dynamical correlation for a well-dressed PEC. For the rest of the molecules, the effect of dynamical correlation is much weaker, e.g., NPE changes from 3 to 0.9 kcal mol^–1^ for C_2_, from 4 to 0.4 kcal mol^–1^ for N_2_, etc. However, all NPE values, even for the B_2_ molecule, are found smaller than the 10 kcal mol^–1^ threshold. The addition of dynamical type connected T, Q, 5, etc., excitations out of the reference function decreases the present NPE values as expected, e.g., the dynamical connected Q and 6 excitations for the N_2_ molecule, etc.

#### Li_2_

**Figure 1:**
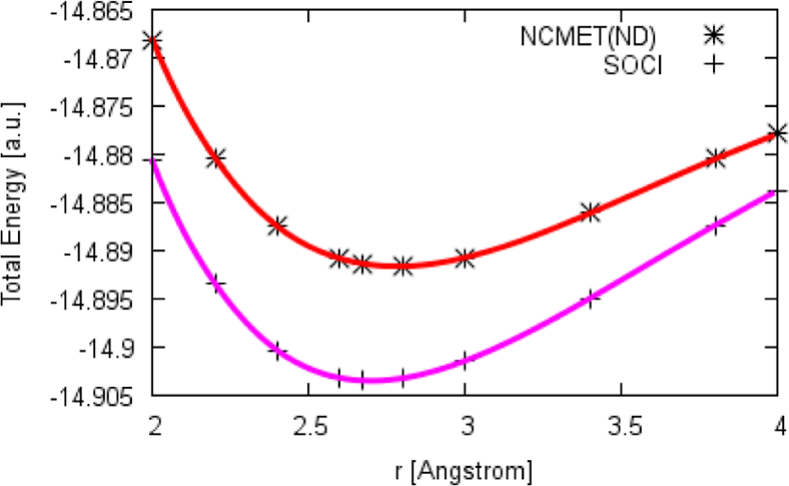
PEC of the Li_2_ molecule in def2-QZVP basis.

**Figure 2:**
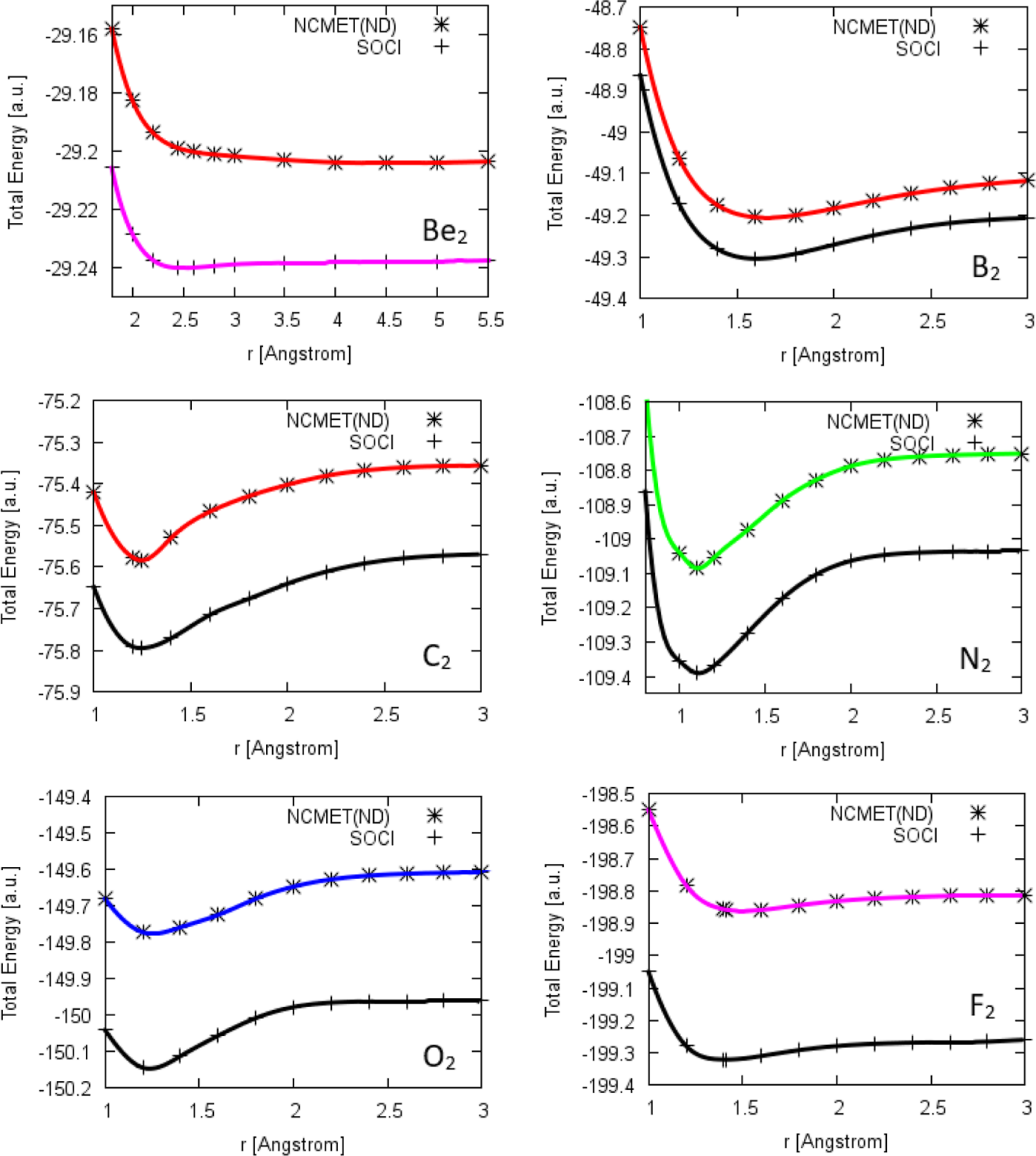
PECs of the studied homonuclear diatomic molecules.

The dilithium molecule is diamagnetic and exists in the vapor phase. Its term symbol is ^1^Σ_g_. The frozen-core full CI calculation for the Li_2_ molecule in augmented 5Z basis gives the total electronic energy as −14.9038041 au corresponding to a correlation energy equal to −0.03226 au. At the CBS(345) limit of augmented nZ basis hierarchy, we estimate the exact valence correlation energy as −0.0324 au. The nondynamical correlation energy of the Li_2_ molecule at the equilibrium geometry calculated from the NCMET(ND) method is predicted to be around −0.02 au which increases to the −0.042 au value at the atomic dissociation limit. The frozen-core full CI energy of the Li_2_ molecule in def2-QZVP basis is −14.9034 au which then gives—after the addition of core correlation—almost −14.9943 au compared to −14.9918 au,^69^ −14.9938 au,^70^ and −14.9952 au^71^ calculated by the diffusion Monte Carlo (DMC) method. However, the exact NR total electronic energy of the Li_2_ molecule is estimated to be −14.9950 au in this work. The PEC of the Li_2_ molecule at the NCMET(ND) and SOCI level of theories was shown in Figure 1 where Mk-MRCCSD and full CI curves coincide with the SOCI curve. The dissociation energy of the dilithium molecule is predicted to be 17.4 kcal/mol by NCMET(ND) and 23.8 kcal/mol by SOCI and MK-MRCCSD methods. The NCMET(ND) and SOCI potential energy curves of the Be_2_–F_2_ sequence are shown in Figure 2. The vibrational wavenumber was calculated to be 356 cm^–1^, compared to the experimental value (351 cm^–1^).

#### Be_2_

**Figure 3:**
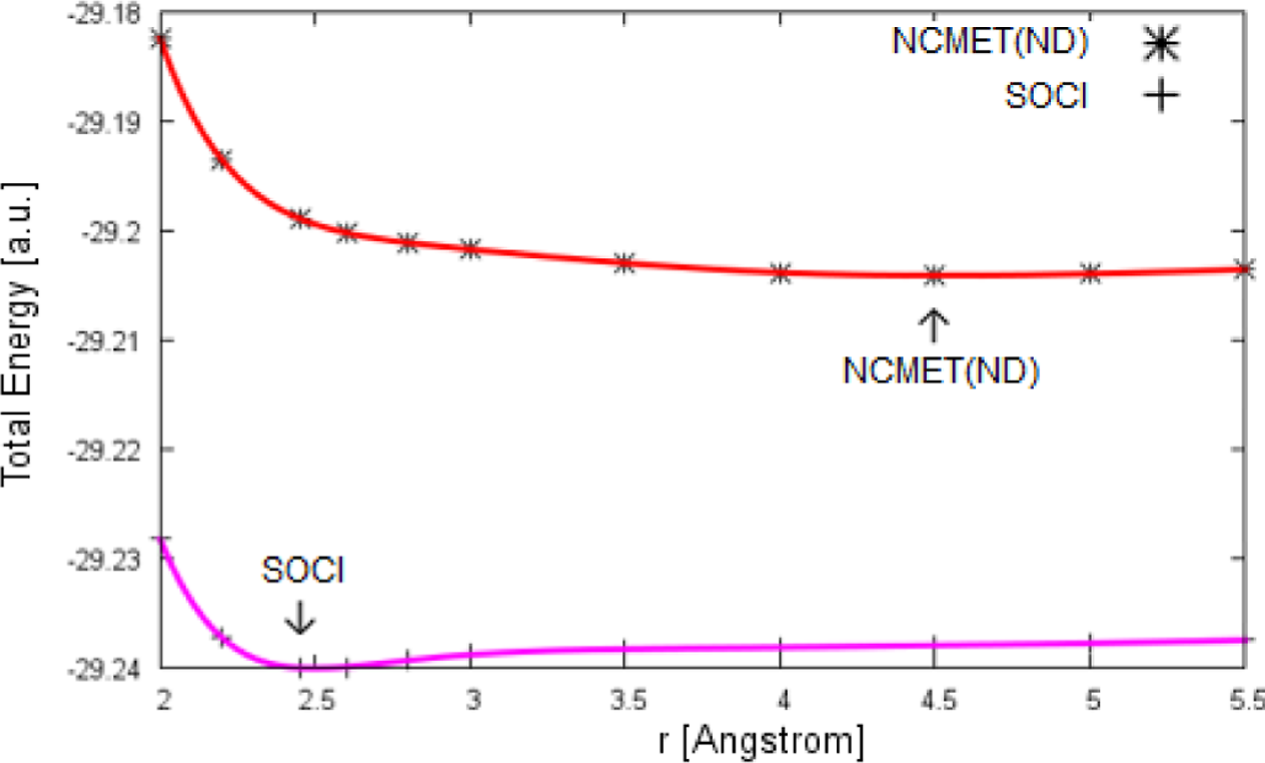
PEC of the Be_2_ molecule. The different minima were shown in detail.

The diberyllium molecule is diamagnetic and exists in the vapor phase. Its term symbol is ^1^Σ_g_. The ability of predicting accurate dissociation energy of a beryllium molecule is a prerequisite for the success of quantum chemical theory or a computational tool. According to Table 5, the dissociation energy in a nonrelativistic, infinite nuclear mass approach in frozen-core approximation is predicted to be 2.47 kcal mol^–1^. Gdanitz^72^ calculated 2.58 kcal mol^–1^ value from nonrelativistic *r*_12_-MR(CAS)-ACPF calculations. Schmidt et al.^73^ predicted 2.25 kcal mol^–1^ from their valCASSCF calculation with some full CI corrections and 2.42 kcal mol^–1^ at the CBS limit. Lesiuk et al.^41^ also reported 2.41 kcal mol^–1^ value which was then extrapolated to 2.47 kcal mol^–1^. On the other hand, the CCSD(T) method underestimates the dissociation energy for the Be_2_ molecule, that is, the author predicts 1.83 kcal mol^–1^ at the CBS limit, i.e., 75% of the nonrelativistic dissociation energy at frozen-core approximation. Even CCSDT is not a sufficient level of theory, which gives 2.28 kcal mol^–1^. All those indicate how important the post-CCSD(T) effects are in the calculation of dissociation energy. Such post-CCSD(T) effects have been taken into account by Patkowski et al.^74^ and a value around 2.45 kcal mol^–1^ may be extracted from their calculations after subtracting relativistic and core correlation corrections. In a recent work,^75^ T–(T) contribution has been estimated in the order of 0.43 kcal mol^–1^ which indicates that CCSD(T) underestimates triple excitations at all geometries for Be_2_. The contribution of connected quadruples (T_4_ cluster) is also calculated as 0.22 kcal mol^–1^, and then the dissociation energy is found to be 2.48 kcal mol^–1^ in the nonrelativistic approach. Figure 3 shows the different minima of the Be_2_ molecule found via NCMET(ND) and SOCI methods. The vibrational wavenumber was calculated to be 264 cm^–1^, compared to the experimental value (278 cm^–1^).

**Figure 4:**
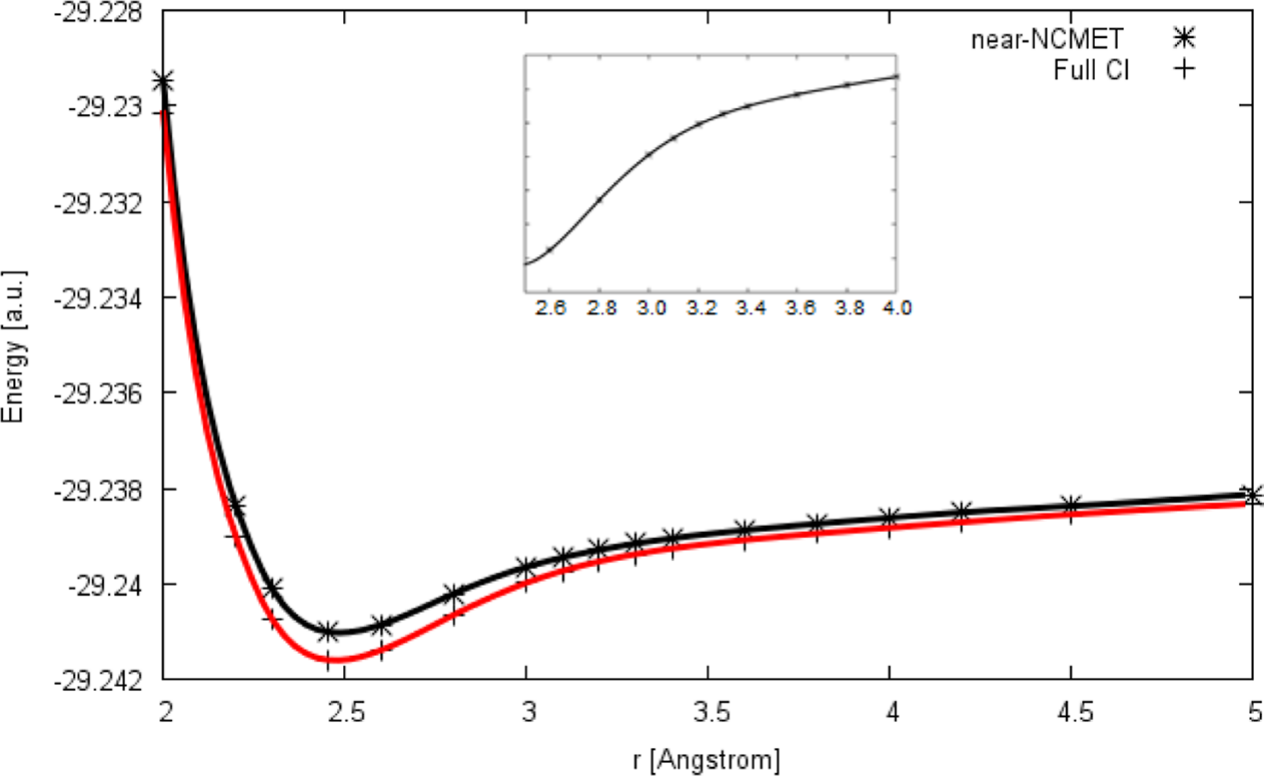
PEC of Be_2_ calculated by near-NCMET and full CI in aug-cc-pVQZ basis (the inset shows the intermediate region of the full CI curve in detail).

An unusual bonding, which is weak by average chemical measures but much stronger than dispersion forces due to its deep potential well, is formed in the Be_2_ molecule inasmuch as the bonding is accompanied entirely by the changes in the nondynamical and dynamical correlations. ROHF and UHF curves do not dissociate to the same asymptote and the molecule has an UHF instability originating from the near-degeneracy of atomic 2s and 2p orbitals—as a part of the internal correlation. The NCMET(ND) wave function still results in a repulsive potential at experimental geometry but gives a shallow well around 4.5 Å which indicates that the dynamical correlation is essential to support bonding at the correct internuclear separation. That is to say, it is well-known that Be_2_ is not bound at the HF level and addition of nondynamical correlation via the NCMET(ND) method predicts a bound system at a large separation; however, it seems it is still insufficient for this molecule to give the minimum at the correct geometry. The minimum of the potential was also predicted to be 5.5 Å by using the CASVB method which includes only a part of the NCMET(ND) wave function. The internal double term (2σ_u_)^2^ → (3σ_g_)^2^ is found prominent in the NCMET(ND) wave function. The addition of dynamical correlation via SOCI and near-NCMET level of theories brings the molecule to the correct point (2.454 Å) on the PES. The SOCI method gives a dissociation energy as 1.63 kcal mol^–1^ in the def2-QZVP basis (and 2.28 kcal mol^–1^ in the CBS limit in the aug-cc-pVXZ hierarchy where X = T, Q, 5). The near-NCMET level of theory gives a deeper minimum at 2.32 kcal mol^–1^ in aug-cc-pVQZ basis and 2.41 kcal mol^–1^ at the CBS limit which corresponds to 99% of the valence contribution to the dissociation energy. Near-NCMET theory also gives 99.6% of the valence correlation energy which is calculated as −0.1077 au at the CBS limit. The near-NCMET potential energy curve appears almost parallel to the full CI curve, Figure 4, and the difference is measured by the nonparallelity error (NPE) which is only of 0.017 kcal mol^–1^.

It is clear that the near-NCMET method predicts the correct asymptotic behavior at the dissociation region. Furthermore, partitioning the dissociation energy into HF and correlation contributions gives us the following values: ΔHF as −7.51, ΔND as 6.30, and ΔD as 3.64 kcal mol^–1^, and then the total correlation contribution is found as 9.94 kcal mol^–1^ for the Be_2_ molecule. Schmidt et al.^73^ also found the same value, but they use a different partition of correlation energy for nondynamical and dynamical correlation which is why the following values are extracted from their calculations as ΔND as 0.95 and ΔD as 8.98 kcal mol^–1^, indicating the same total value as 9.93 kcal mol^–1^. Although the contribution of nondynamical correlation to the dissociation energy of the beryllium molecule is almost twice that of dynamical correlation, nondynamical correlation alone cannot indicate the ground state energy minimum, and dynamical correlation is requisite for this molecule. valCAS-DMC type calculation^76^ in QZ basis gives 1.64 kcal mol^–1^ which is almost identical to SOCI calculation here, both in types of excitations included and also in the predicted value as discussed above. By using a larger CAS space with 16 orbitals in the DMC procedure, i.e., by expanding the CAS space to include MOs derived from 3s and 3p AOs, the dissociation energy by the CAS(4,16)-DMC method was predicted as 2.34 kcal mol^–1^; now this is identical to the near-NCMET result in augmented QZ basis as reported in this work. For the Be_2_ molecule, it is sometimes reported that a substantial change in the slope around 3.2 Å separation occurs.^73^ In our full CI calculations in relatively large and balanced aug-cc-pVQZ basis, such a change in the slope is not found, but a smooth curve is generated, Figure 4. Following the present NCMET calculations, it is found that the valence correlation energy (−0.1081 au) at *R*_e_ increased to −0.1238 au value at the dissociation region. Both types of correlations are found increased, i.e., ND correlation increased by −0.01 au and D correlation by −0.005 au; however, their percentage in the valence correlation energy remained the same. The frozen-core full CI energy of the Be_2_ molecule in def2-QZVP basis is −29.2399 au (compared to −29.24158 au from frozen-core MRCI calculation in QZ basis^77^) which then gives—after the addition of core correlation—almost −29.3366 au compared to −29.3301 au^70^ and −29.3336 au^71^ calculated by the DMC method and −29.3377 au^78^ by CCSD(T) at the CBS limit. However, the exact NR total electronic energy of the Be_2_ molecule is estimated as −29.3390 au in this work.

#### B_2_

The diboron molecule has paramagnetic behavior, and it exists in the vapor phase. Its term symbol is ^3^Σ_g_. The nondynamical correlation energy of the B_2_ molecule at its equilibrium geometry calculated from the NCMET(ND) method (−0.126 au) increases to a value around −0.2 au at the atomic dissociation limit. The change in the internal and semi-internal correlation energies is found to be around 0.045 and 0.035 au, respectively, which indicates that both types of nondynamical correlation almost equally contribute to dissociation energy. As a triplet ground state molecule, the semi-internal correlation plays a crucial role in molecular properties of B_2_. The same is also true for the dioxygen molecule. In the case of the B_2_ molecule, the convergence of SOCI energy is found somewhat slower. The bias of the core–valence basis sets toward describing core–core correlation over core polarization may be put forward as the reason for this. Figure 2 shows the PEC of B_2_ calculated by the NCMET(ND) and SOCI methods. Mk-MRCCSD(T) and SOCI methods predict the dissociation energy of diboron as 64.0 and 65.4 kcal mol^–1^, respectively. Near-NCMET predicts the dissociation energy to be 65.9 kcal/mol in QZ basis. The expectation values of the XX and ZZ components of the electric quadrupole moment of the B_2_ molecule calculated by the NCMET(ND) method are relatively smaller than the ones calculated by the SOCI method which also indicates the necessity of dynamical correlation for the diboron molecule, Table S2. The calculated vibrational wavenumber value (1089 cm^–1^) also supports the importance of dynamical correlation for diboron (exp. value is 1051 cm^–1^, MRCI value including dynamical correlation is 1039 cm^–1^, from ref (30)). However, the expectation values of relativistic terms are almost equal to each other, Table S2. The other molecules for which the dynamical correlation is necessary for the calculation of quadrupole moment are Li_2_ and Be_2_ molecules.

#### C_2_

The dicarbon molecule has diamagnetic behavior and it exists in the vapor phase. Its term symbol is ^1^Σ_g_. The ground state of the dicarbon molecule exhibits an unusual bonding which is depicted as a “nearly empty sandwich” of a double bond. The internal double term (2σ_u_)^2^ → (3σ_g_)^2^ is found to be very significant and plays a secondary role in the doubly bonded C_2_ molecule. The presence of this configuration suggests that this double bond may be stronger than it appears when only the single reference function is considered. Unlike its canonical doubly bonded structure, triply^79^ and quadruply^80^ bonded structures were also proposed; on the other hand, a sigma-bonded structure along with the remaining valence electrons as antiferromagnetically coupled^67^ has also been proposed. However, it was also suggested that none of these bonding schemes allows one to give the equilibrium structure.^81^ In the present work, NCMET(ND) expansion, which includes excitations up to octuples, i.e., NCMET(ND)SDTQ5678, predicts the dissociation energy to be 140 kcal mol^–1^, corresponding to 95% of the NR(FC) energy value. The valCASSCF method and SCAN meta-GGA functional^82^ both give 143 kcal mol^–1^. For a comparison, the dissociation energy was also calculated from the CASVB method, which predicts 143.4 kcal/mol both in def2-QZVP and in augmented polarized valence QZ basis sets, but its PEC diverges after 4.5 Å, likewise the valCASSCF curve. At this juncture, it is worth stressing that one predicts 147 kcal mol^–1^ from NCMET(ND) expansion when SDTQ type correlations are included only, i.e., NCMET(ND)SDTQ, while NCMET(ND) expansion with SDTQ56 correlations, i.e., NCMET(ND)SDTQ56, gives 142 kcal mol^–1^. It is obvious that pentuple and hextuple excitations are essential for a well-dressed potential energy curve with a lower NPE value, but triple and quadruple excitations seem to be enough to predict the breaking of the “double” bond—the PEC at the SDTQ level is also smooth through 8 Å—and that is why the rest of the triple and quadruple bonding assertions may still be attributed to the remaining correlation effects rather than some complex bonding schemes. A recent experiment on the photoelectron spectrum of dicarbon attributed the unusual character of the carbon–carbon double bond to the unique nature of two pi bonds with no σ bond and suggested that the triple and the quadruple bond configurations only have a small influence on the overall bonding nature.^83^Figure 2 shows the PEC of C_2_ calculated by the NCMET(ND) and SOCI methods.

In the case of the C_2_ molecule, the increase in the nondynamical correlation energy from equilibrium separation to the dissociation region is found to be the largest in the Li_2_–F_2_ sequence. It is almost found doubled (the change is in the order of −0.2 au) and followed by the increase in the nondynamical correlation energy for N_2_ (around −0.15 au) and O_2_ (around −0.13 au). This result is also parallel to the MR nature of these molecules. The internal correlation in the C_2_ molecule also becomes more important at the distances starting at 1.5 Å where the CCSD(T) curve starts to deviate from the full CI curve. It is not surprising at this point that both CCSD(T) and CCSDT curves exhibit a divergent behavior right after 2 Å,^84^ even before the 2*R*_e_ intermediate region. The inclusion of connected quadruples is essential in this case, and the nondynamical portion of it is already included in the NCMET(ND) wave function. The contribution of pentuple excitations was found to reduce the NPE value only moderately. This is consistent with the fact that the odd-tuply excitations cause only a modest improvement of the results.^85,86^ Upon addition of the dynamical correlation via the near-NCMET method, the dissociation energy is predicted to be 144 kcal/mol in QZ basis. The vibrational wavenumber was also calculated in the same basis and found as 1874 cm^–1^, compared to the experimental value (1855 cm^–1^).

#### N_2_

The dinitrogen molecule is diamagnetic and exists in the vapor phase in the standard state. Its term symbol is ^1^Σ_g_. In contrast to other diatomics in the series, the semi-internal correlation is found mostly canceled upon dissociation of the N_2_ molecule; that is why the dissociation energy of dinitrogen is mostly determined via the change in the internal correlation energy. The nondynamical correlation energy of the N_2_ molecule at its equilibrium separation (−0.123 au) increases to −0.272 au value at the dissociation limit. Figure 2 shows the PEC of N_2_ calculated by the NCMET(ND) and SOCI methods. The expectation values of the XX and ZZ components of the electric quadrupole moment of the N_2_ molecule calculated by NCMET(ND) and SOCI methods are consistent with each other, Table S2. The scalar relativistic terms calculated by the NCMET(ND) method are also close to the SOCI ones. In addition, the vibrational wavenumber was predicted to be 2394 cm^–1^, compared to the experimental value (2377 cm^–1^).

#### O_2_

The dioxygen molecule has a very well-known paramagnetic behavior. It exists in the vapor phase in a standard state, and its term symbol is ^3^Σ_g_. Among double correlation terms in the NCMET(ND) wave function of the O_2_ molecule, the internal double term (1π_u_)^2^ → (1π_g_)^2^ is found to be very significant that produces the (core)(2σ_g_)^2^(2σ_u_)^2^(3σ_g_)^2^(1π_u_)^2^(1π_g_)^4^ configuration. The internal quadruple term (3σ_g_)^2^(1π_u_)^2^ → (3σ_u_)^2^(1π_g_)^2^ is especially found prominent for the O_2_ molecule. This term is also related to the fact that why 3σ_g_ and 3σ_u_ orbitals become degenerate at the long-range region. The polarization terms, e.g., 1π_u_ → 2π_u_, etc., are important at large separations as well. The nondynamical correlation is calculated as −0.128 au, most of which is due to semi-internal correlation in comparison to the other diatomics in this series. The frozen-core i-FCIQMC method, which is also not size-consistent, predicted the dissociation energy as 117.5 kcal mol^–1^ in QZ basis^87^ in comparison to the SOCI value around 116 kcal mol^–1^. This method gives the dissociation energies of C_2_, N_2_, and F_2_ almost identical to the SOCI values of this work, as well. Figure 2 shows the PEC of O_2_ calculated by the NCMET(ND) and SOCI methods. The related vibrational wavenumber was calculated to be 1631 cm^–1^ (exp. value 1610 cm^–1^).

#### F_2_

The difluorine molecule is diamagnetic and exists in the vapor phase in the standard state. Its term symbol is ^1^Σ_g_. The PEC of the F_2_ molecule has been a popular benchmark for testing electron correlation methods for a long time. The dissociation of difluorine is a rather challenging example due to strong correlation effects as both nondynamical and, mostly, dynamical. The quasi-degeneracy effect is also important.^88^ It is well-known that difluorine is unbound at the HF level of theory and also one of the weakest covalently bound species. Addition of the internal correlation type (3σ_g_)^2^ → (3σ_u_)^2^ double excitation term to the ground state wave function is essential to predict the correct sign of dissociation energy which is found to be 16 kcal mol^–1^. The dissociation energy calculated at the NCMET(ND) level of theory predicts almost 90% of the correct value. In this wave function, (3σ_g_)(1π_g_) → (3σ_u_)(2π_u_) and (3σ_g_)(1π_u_) → (3σ_u_)(2π_g_) type semi-internal double excitations are found crucial for proper dissociation. The calculations including noniterative triples for the F_2_ molecule revealed once again that the perturbative inclusion of noniterative triple correlations does not always fit well to the correct asymptotic character of PEC.^89^ A balanced inclusion of triple excitations seems to be essential in the long-range region of the PECs. In SOCI and near-NCMET calculations, the dynamical type (3σ_g_)^2^(1π) → (4σ_g_)(5σ_g_)(3π) and (3σ_g_)^2^(1π) → (4σ_g_)(6σ_g_)(3π) triple excitation set is found prominent that should also be noted. It is also found that the nondynamical correlation energy at *R*_e_ (−0.1141 au) increased to −0.207 au value at the dissociation region. The change in the internal correlation energy is found dominant (the change is in the order of 0.073 au) in determining the dissociation energy. This is not surprising because of the near-degeneracy of 3σ_g_ and 3σ_u_ orbitals at stretched geometries beyond 4.5 Å (whereas single reference methods, e.g., CCSD(T), fail even before reaching 3 Å). However, the semi-internal correlation is largely canceled and contributes in the order of 0.02 au. The vibrational wavenumber was calculated to be 885 cm^–1^, compared to the experimental value (908 cm^–1^). As compared to the MRCI value (896 cm^–1^) from ref (30), the dynamical correlation was found significant for difluorine as well as for diboron.

**Figure 5:**
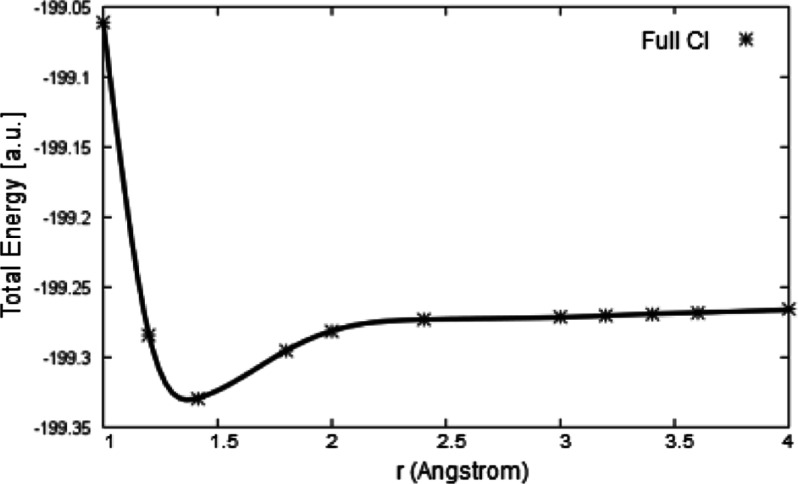
Full CI PEC of the F_2_ molecule in aug-cc-pVQZ basis (calculated within 2 weeks in real time).

In previously reported values that were calculated by single reference CC and MPn methods—a survey of these results may be found in ref (22)—the dissociation energy has been calculated from the part of the PEC near equilibrium (almost around 1.5*R*_e_ or 2*R*_e_), that is because the PECs computed by SR methods like MP4, CCSD, CCSD(T), etc., start to deviate right after 2*R*_e_ separation and completely diverge. The MR-BWCCSD method with an a posteriori size-extensivity correction gives the dissociation energy as 31.6 kcal mol^–1^ in the cc-pVQZ basis. The remaining energy which is almost 7.5 kcal mol^–1^ is about the lack of triple excitations in the cluster expansion and the need for a larger active space. In SOCI calculations, the QZ basis is also found sufficient and the dissociation energy is predicted as 33.7 kcal mol^–1^. This is also in accordance with the previously reported literature results.^22,30^ In the aug-cc-pVnZ hierarchy of basis sets, the SOCI/CBS level of theory gives 35 kcal mol^–1^ and the rest of the 4 kcal mol^–1^ energy may be attributed to the lack of some part of higher dynamical correlations near *R*_e_. Bytautas et al.^90^ predicted that the PEC of F_2_ shows a repulsive character over the 2*R*_e_ region only when the internal correlation has been taken into account and gives a hump (0.01–0.1 eV) around 2.9–3 Å; however, they concluded that the inclusion of the dynamical correlation effects overcomes this repulsive character. The same nonphysical hump has also been found before by Mášik et al.^91^ from MR-MBPT2 and CASPT2 calculations and also by Csontos et al.^42^ from MRCI and MRCC calculations. In the present work, the NCMET(ND) wave function which includes whole nondynamical correlation (the sum of internal correlation, semi-internal correlation, and polarization effects) does not predict this hump and gives a smooth PEC, Figure 2. Adding dynamical correlation produces a parallel PEC to the former, i.e., SOCI PEC is smooth, does not possess any hump, and displays a correct long-range behavior. This also confirms the necessity of dynamical correlation such that the correct *D*_e_ value cannot be found without it. The state-specific MRCCSD calculations^92^ also support this conclusion about the absence of such a nonphysical hump. No barrier or hump has also been observed in some previous works.^40,93−96^ A fictitious barrier was observed in a CIPSI calculation in DZ basis with 1000 determinants and it was associated with the lack of convergence; however, this artifact disappeared after the number of determinants exceeded 5000.^97^ To make a conclusive investigation, the full CI potential energy curve of F_2_ in aug-cc-pVQZ basis is also calculated in this work, Figure 5.

The nondynamical and dynamical type correlations higher than double excitations are also considered rigorously. The contributions that are predicted from NCMET(ND), CISDTQ, CISDTQ56, and CISDTQ78 calculations are shown in Table 8.

**Table 8:** ND Type and Total (ND and D) Contributions of Correlation Types Higher than Doubles to Valence Correlation Energy in def2-QZVP Basis (in au)

		ND	ND + D^a^
Be_2_	TQ	–0.0016	–0.0108 (−0.01093)
B_2_	TQ	–0.0040	–0.0263 (−0.02700)
	56	∼0	–0.0006 (−0.00069)
C_2_	TQ	–0.0089	–0.0631 (−0.07191)
	56	–0.0002	–0.0066 (−0.00877)
	78	∼0	(−0.00021)
N_2_	TQ	–0.0014	–0.038 (−0.04871)
	56	∼0	(−0.00333)
	78	0	(−0.00008)
O_2_	TQ	–0.0002	–0.031 (−0.04370)
	56	0	(−0.00175)
	78	0	(−0.00002)
F_2_	TQ	–0.0002	–0.043 (−0.06455)
	56	0	(−0.00381)
	78	0	(−0.00006)

a The values in parentheses are estimations at the CBS limit,^46^ except Be_2_ and B_2_ (this work).

It is clear to conclude that 15% of valence TQ correlations is of nondynamical type for Be_2_, B_2_, and C_2_ molecules. This contribution decreases to 3% for N_2_ where the electrons are more densely packed and only 1% for O_2_ and F_2_ molecules. Nondynamical type 56 correlations correspond to 3% of the total 56 correlation energies in the C_2_ molecule, and in total, almost 17% of higher correlations (TQ5678) are of nondynamical type correlation. In addition to this, 56 correlations in N_2_, O_2_, and F_2_ and 78 correlations in C_2_ are found fully in dynamical character. Despite the fact that they do not contribute to the valence correlation energy, the nondynamical type 5678 correlations are essential to ameliorate the wave function at the long-range part of the PEC which is why they are included in NCMET(ND) wave functions. Of course, these TQ5678 correlations are discussed here in CI language, e.g., Qs include both connected and disconnected quadruples, etc. The contribution of connected clusters, such as T_3_, T_4_, T_5_, etc., is commonly estimated from CCSDTQ, CCSDTQ5, etc., calculations.^14^ The effect of connected quadruples (T_4_) is the highest for the C_2_ molecule (−0.0037 au), which corresponds to 8% of total quadruples. It is followed by the B_2_ molecule (−0.002 au) which is lower compared to T_4_ correlations in C_2_; however, it corresponds to 20% of total quadruples in B_2_. The contribution of the T_4_ term was estimated as 20% of total quadruples for B_2_, around 10% for C_2_ and O_2_, and 5% for N_2_ and F_2_ molecules. The total contribution of T_4_ and T_5_ terms was estimated at around 20–25% for B_2_, C_2_, and O_2_ and 15% for N_2_ while it is found around 10% for the F_2_ molecule.^98^ Feller et al.^99^ also estimated the FCI corrections to CCSDTQ dissociation energies by making use of the continued fraction (cf) approximant, and especially, for diboron, it was estimated to be as large as the corrections for dinitrogen and dioxygen and larger than difluorine. Their findings also support our conclusion on the importance of dynamical type higher-order correlation effects for the diboron molecule.

In the case of Li_2_ and Be_2_ molecules, the CI expansion is short compared to B_2_–F_2_ systems. As a final remark, the norm of the CI vector, ||*X*||_2_, along with the sum of the absolute values of the coefficients, ||*X*||_1_, is also calculated to investigate the dispersity of small coefficients in the NCMET(ND) type MCCI vector. These quantities are defined as
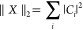
7
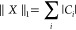
8where ||*X*||_2_ norm is almost unity in all cases. ||*X*||_1_ values of the CI vectors are calculated as 5.005 (B_2_), 4.128 (C_2_), 2.941 (N_2_), 3.299 (O_2_), and 1.884 (F_2_), respectively. The number of coefficients reaches its maximum in the 10^–3^ class, mostly in the case of the B_2_ molecule. The number of small coefficients (10^–5^ and 10^–6^) is small, as expected. It reaches its maximum in the 10^–5^ class. The ||*X*||_1_ value is much more dispersed for B_2_ molecules, which indicates that a relatively large number of dynamical correlation terms are required to obtain an accurate CI vector. This is also revealed in the magnitude of the components of the quadrupole moment of the B_2_ molecule.

The calculated values of the relativistic correction and the ZZ component of the electric quadrupole moment of these molecules are also summarized in Table 9. The other components for both expectation values of relativity and quadrupole moment terms are given in Table S2 in detail. It seems to be remarked that for B_2_ molecules, the NCMET(ND) method gives relatively smaller quadrupole moment values than the ones calculated by the SOCI method, which also indicates the necessity of dynamical correlation for diboron again. However, the expectation values of relativistic terms are almost equal to each other, Table S2. The other molecules for which the dynamical correlation is necessary for the calculation of quadrupole moment are Li_2_ and Be_2_ molecules. The electric quadrupole moment of the N_2_ molecule calculated by NCMET(ND) and SOCI methods is consistent with each other. The scalar relativistic terms calculated by the NCMET(ND) method are also close to SOCI ones.

**Table 9:** Relativistic Corrections (au) and the Quadrupole Moments (in D Å)

		ROHF	NCMET(ND)	SOCI
Li_2_	Rel.	–0.001352	–0.001350	–0.001352
	Θ_ZZ_	10.61	9.02	10.81
Be_2_	Rel.	–0.005024	–0.005001	–0.005006
	Θ_ZZ_	–3.68	–3.64	–3.10
B_2_	Rel.	–0.013467	–0.013402	–0.013408
	Θ_ZZ_	1.17	0.40	0.83
C_2_	Rel.	–0.029858	–0.029727	–0.029722
	Θ_ZZ_	2.80	2.23	2.26
N_2_	Rel.	–0.058277	–0.058242	–0.058457
	Θ_ZZ_	–0.98	–1.13	–1.19
O_2_	Rel.	–0.104142	–0.104085	–0.104489
	Θ_ZZ_	–0.30	–0.44	–0.32
F_2_	Rel.	–0.174138	–0.174128	–0.174660
	Θ_ZZ_	0.51	0.58	0.68

## Conclusions

In the present work, the nondynamical correlation energies of first row atoms and their homonuclear diatomic molecules were calculated by using the MCCI type NCMET(ND) method. The dynamical correlation energies were then calculated by MRCI type SOCI and near-full NCMET and MRCC type MK-MRCCSD(T) methods and compared to the full CI values, which were also calculated for the whole series. The contributions of nondynamical and dynamical correlations to dissociation energies were rigorously investigated. The effects of core correlation, scalar relativistic, and spin–orbit and the effects beyond the adiabatic approximation were all taken into account. The contribution of dynamical correlation to dissociation energy was found to be less than 10% except for the dilithium molecule (25%). The contribution of nondynamical correlation was found to be equal or more than 70% for B_2_, C_2_, and O_2_ molecules. For dinitrogen, the HF orbital part contributes more than 50% which is why the contribution of nondynamical correlation is in the order of 40% for this system. In the case of the Be_2_ molecule, it is found that the NCMET(ND) wave function must be ameliorated by the addition of dynamical correlation. An in-depth analysis of the Be_2_ curve showed the significance of the dynamical triple excitations to yield the correct shape of the PEC.

Almost the whole binding is of ND correlation for B_2_ and C_2_; some authors only consider internal correlation as ND type correlation and that is why they attribute some important portion of ND correlation effects to dynamical correlation. The proper use of ND correlation as the sum of internal and semi-internal correlations was also found parallel to the MR characters of the molecules and consistent with the related MR diagnostics. The dynamical correlation with large basis sets is recognized as important elements for the PEC of the F_2_ molecule. Although the difluorine molecule has a lower multireference character than B_2_, C_2_, and N_2_ molecules, it has a unique strangeness. Both semi-internal and external triple correlations are found to be strongly dominant; however, even after including a balanced triple contribution, the difluorine system is still a hard problem to be treated at the long range. This is not purely of the dynamical correlation but the multireference character of the molecule at the long-range region of PEC due to the near-degeneracy of orbitals involved and the large number of LP–LP interactions. All those reflect the necessity of higher correlation terms like connected quadruple, connected pentuple, etc., in both nondynamical and dynamical types.

Although the NCMET(ND) method works well in treating PECs and corrects the HF wave function for the multireference character of the problem, the dynamical correlation is still essential to decorate the wave function for an accurate prediction of chemical properties and also to obtain PECs of spectroscopic quality. The near-NCMET results, which are calculated for this purpose along with SOCI results in many cases, agree well with the current state-of-the-art calculations. Especially, near-NCMET results of the present work are close to full CI quality and reflect the power of the theory, not only theoretically but also computationally. The SOCI method, a simpler but more robust one, with respect to computational efficiency and memory requirements, also provides us with a balanced description of the potential energy curve with a correct shape over the whole internuclear distances. Full CI calculations along with CIPSI results were also performed where possible. Full CI results with a large and balanced basis set, such as aug-cc-pVQZ, also reflect the accuracy of the theoretical data. It is found that the further extension of the basis set beyond QZ quality would not bring any substantial improvement on the NCMET (or SOCI) results; however, the only reasonable extension is about the inclusion of triple excitations out of NCMET(ND) (and also CAS) reference. The present results demonstrate that the ground states are well-represented by the near-NCMET theory, with a NCMET(ND) reference function constructed over SCF orbitals. SOCI results with a valCASCI reference function constructed over SCF orbitals also show good quality. To conclude, the near-NCMET method is shown to provide an accurate and consistent description of nondynamical and dynamical correlation relying upon the balanced and compact NCMET(ND) wave function by handling a tiny fraction of the whole Hilbert space. This work suggests that with continued development, the near-NCMET level of full NCMET theory may be expected to provide valuable insight into a range of relatively larger systems. The performance of NCMET(ND) was also tested for the one-electron properties such as the expectation values of scalar relativistic terms and electric quadrupole moments. The results were found consistent with the ones produced by the SOCI method and also the available experimental quadrupole moment data.

## Supporting Information Available

Additional data on the expectation values, the list of configurations in the wave functions, and sample input text (PDF)
