# Supplementary material for: Multiconfigurational Study on the Contribution of the Nondynamical and Dynamical Correlation Energies to the Dissociation Energies of Li2-to-F2 Molecules
Source: ACS Omega. 2025 Apr 28;10(18):18848–63. doi: 10.1021/acsomega.5c00734 (PMC12079217; doi:10.1021/acsomega.5c00734)
Supplement: Supplementary file 1 — ao5c00734_si_001.pdf [file ao5c00734_si_001.pdf]

# SUPPORTING INFORMATION

## A Multi-Configurational Study on The Contribution of The Nondynamical and Dynamical Correlation Energies to The Dissociation Energies of $Li_2$-to-$F_2$ Molecules

Berkay Sütay

Istanbul Technical University, Department of Chemistry, Istanbul.

## A. TABLES

**Table S1**. Relativistic and spin-orbit corrections (kcal/mol) to dissocitation energies calculated by NCMET (ND) and SOCI in def2-QZVP basis.

| | Δrel (NCMET) | Δrel (SOCI) | ΔSO (CISD) | ΔSO (exp) |
|---|---|---|---|---|
| $Li_2$ | -0.0003 | - | 0 | 0 |
| $Be_2$ | -0.011 | -0.012 | 0 | 0 |
| $B_2$ | -0.054 | -0.060 | -0.05 | -0.06 |
| $C_2$ | -0.140 | -0.176 | -0.17 | -0.17 |
| $N_2$ | -0.197 | -0.134 | 0 | 0 |
| $O_2$ | -0.290 | -0.186 | -0.45 | -0.4 |
| $F_2$ | -0.112 | -0.036 | -0.76 | -0.8 |

**Table S2.** The expectation values of relativistic corrections to total electronic energy (a.u.) and the components of quadrupole moment (in D.Å) at different levels of theories.

| | | ROHF | NCMET(ND) | SOCI |
|---|---|---|---|---|
| $Li_2$ | MV | -0.008196 | -0.008186 | -0.008191 |
| | Darwin | 0.006843 | 0.006836 | 0.006839 |
| | Rel. | -0.001352 | -0.001350 | -0.001352 |
| | $\Theta_{XX}$ | -5.30 | -4.50 | -5.40 |
| | $\Theta_{ZZ}$ | 10.61 | 9.02 | 10.81 |
| $Be_2$ | MV | -0.028369 | -0.028285 | -0.028292 |
| | Darwin | 0.023345 | 0.023284 | 0.023286 |
| | Rel. | -0.005024 | -0.005001 | -0.005006 |
| | $\Theta_{XX}$ | 1.84 | 1.82 | 1.53 |
| | $\Theta_{ZZ}$ | -3.68 | -3.64 | -3.10 |
| $B_2$ | MV | -0.072730 | -0.072487 | -0.072446 |
| | Darwin | 0.059262 | 0.059085 | 0.059037 |
| | Rel. | -0.013467 | -0.013402 | -0.013408 |
| | $\Theta_{XX}$ | -0.58 | -0.20 | -0.41 |
| | $\Theta_{ZZ}$ | 1.17 | 0.40 | 0.83 |
| $C_2$ | MV | -0.155859 | -0.155350 | -0.155107 |
| | Darwin | 0.126001 | 0.125623 | 0.125385 |
| | Rel. | -0.029858 | -0.029727 | -0.029722 |
| | $\Theta_{XX}$ | -1.40 | -1.12 | -1.13 |
| | $\Theta_{ZZ}$ | 2.80 | 2.23 | 2.26 |

| | | | | |
|---|---|---|---|---|
| $N_2$ | MV | -0.295680 | -0.295586 | -0.295848 |
| | Darwin | 0.237403 | 0.237344 | 0.237391 |
| | Rel. | -0.058277 | -0.058242 | -0.058457 |
| | $\Theta_{XX}$ | 0.49 | 0.57 | 0.60 |
| | $\Theta_{ZZ}$ | -0.98 | -1.13 | -1.19 |
| $O_2$ | MV | -0.515026 | -0.514782 | -0.515477 |
| | Darwin | 0.410884 | 0.410697 | 0.410988 |
| | Rel. | -0.104142 | -0.104085 | -0.104489 |
| | $\Theta_{XX}$ | 0.15 | 0.22 | 0.16 |
| | $\Theta_{ZZ}$ | -0.30 | -0.44 | -0.32 |
| $F_2$ | MV | -0.839946 | -0.840026 | -0.840664 |
| | Darwin | 0.665808 | 0.665898 | 0.666004 |
| | Rel. | -0.174138 | -0.174128 | -0.174660 |
| | $\Theta_{XX}$ | -0.25 | -0.29 | -0.34 |
| | $\Theta_{ZZ}$ | 0.51 | 0.58 | 0.68 |

## B. THE LIST OF THE DETERMINANTS INCLUDED IN NCMET(ND) WAVE FUNCTION

The determinantal functions below are based on the following order of orbitals:

for $Li_2$, $Be_2$, $B_2$ and $O_2$:
$2\sigma_g 3\sigma_g 4\sigma_g 5\sigma_g 6\sigma_g 7\sigma_g 1\pi_{ux} 2\pi_{ux} 3\pi_{ux} 4\pi_{ux} 1\pi_{uy} 2\pi_{uy} 3\pi_{uy} 4\pi_{uy} 1\delta 2\delta 2\sigma_u 3\sigma_u 4\sigma_u 5\sigma_u 6\sigma_u 7\sigma_u 1\pi_{gx} 2\pi_{gx} 3\pi_{gx} 1\pi_{gy} 2\pi_{gy} 3\pi_{gy} 1\varphi 2\varphi$

for $C_2$, $N_2$ and $F_2$:
$2\sigma_g 3\sigma_g 4\sigma_g 5\sigma_g 6\sigma_g 7\sigma_g 8\sigma_g 1\pi_{ux} 2\pi_{ux} 3\pi_{ux} 4\pi_{ux} 1\pi_{uy} 2\pi_{uy} 3\pi_{uy} 4\pi_{uy} 1\delta 2\delta 2\sigma_u 3\sigma_u 4\sigma_u 5\sigma_u 6\sigma_u 7\sigma_u 8\sigma_u 1\pi_{gx} 2\pi_{gx} 3\pi_{gx} 1\pi_{gy} 2\pi_{gy} 3\pi_{gy} 1\varphi 2\varphi$

**Li2 molecule NCMET(ND) WF**
200000000000000000000000000000
000000200000000000000000000000
000000000020000000000000000000
000000000000000020000000000000
020000000000000000000000000000
000000000000000002000000000000
000000000000000000000020000000
000000000000000000000000020000
110000000000000000000000000000
101000000000000000000000000000
100100000000000000000000000000
100010000000000000000000000000
100001000000000000000000000000
011000000000000000000000000000
010100000000000000000000000000
010010000000000000000000000000
010001000000000000000000000000
000000110000000000000000000000
000000101000000000000000000000
000000100100000000000000000000

000000000011000000000000000000
000000000010100000000000000000
000000000010010000000000000000
000000000000000011000000000000
000000000000000010100000000000
000000000000000010010000000000
000000000000000010001000000000
000000000000000010000100000000
000000000000000000000011000000
000000000000000000000010100000
000000000000000000000000011000
000000000000000000000000010100

**Be2 molecule NCMET(ND) WF**
200000000000000020000000000000
200000000000000020000000000000
220000000000000000000000000000
200000200000000000000000000000
200000000020000000000000000000
000000000020000020000000000000
000000200000000020000000000000
100000100000000010000010000000
100000000010000010000000010000
200000000000000000000020000000
200000000000000000000000020000
110000000000000011000000000000
020000000000000020000000000000
000000000000000022000000000000
000000000000000020000000020000
000000000000000020000020000000
200000000000000002000000000000
011000000000000020000000000000
110000000000000010100000000000
000000000011000020000000000000
000000110000000020000000000000
200000000011000000000000000000
200000110000000000000000000000
100100000000000011000000000000
211000000000000000000000000000
101000000000000011000000000000
100000000001000010000000010000
100000010000000010000010000000
000000000000000021010000000000
200000000000000001010000000000
010100000000000020000000000000
000000000000000020000011000000
000000000000000020000000011000
200000000000000000000000011000
200000000000000000000011000000
200000000000000001100000000000
000000000000000021100000000000

020000200000000000000000000000
020000000020000000000000000000
000000000020000000000000020000
000000200000000000000002000000
000000200020000000000000000000
020000000000000002000000000000
020000000000000000000000020000
020000000000000000000002000000
110000000000000000000000020000
110000000000000000000002000000
000000000020000000000002000000
000000200000000000000000020000
000000100010000000000010010000
110000000020000000000000000000
110000200000000000000000000000
010000000010000001000000010000
010000100000000001000010000000
000000000000000011000000020000
000000000000000011000020000000
000000000000000000000020020000
110000000011000000000000000000
110000110000000000000000000000
120100000000000000000000000000
110000000000000001100000000000
121000000000000000000000000000
000000110000000001100000000000
000000000011000011000000000000
020000000000000010010000000000
020000000000000010100000000000
020000110000000000000000000000
020000000011000000000000000000
110000000000000010010000000000
110000000000000010001000000000
110000000000000010000100000000
000000000010100020000000000000
000000000010010020000000000000
000000101000000020000000000000
000000100100000020000000000000
200000000010100000000000000000
200000000010010000000000000000
200000101000000000000000000000
200000100100000000000000000000
210100000000000000000000000000
210010000000000000000000000000
210001000000000000000000000000
100000000000100010000000010000
100000000000010010000000010000
100000001000000010000010000000
100000000100000010000010000000
000000000000000020000010100000
000000000000000020000000010100
200000000000000000000000010100

200000000000000000000010100000
000000000000000021001000000000
000000000000000021000100000000
010010000000000020000000000000
010001000000000020000000000000
100010000000000011000000000000
100001000000000011000000000000
200000000000000010010000000000
200000000000000010001000000000
110000000000000020000000000000
101000000000000020000000000000
100100000000000020000000000000
100010000000000020000000000000
100001000000000020000000000000

**B2 molecule NCMET(ND) WF**

200000100010000020000000000000
220000100010000000000000000000
210000000010000010000010000000
210000100000000010000000010000
200000000000000020000010010000
200000100010000000000020000000
200000100010000000000000020000
100000100020000010000000010000
100000200010000010000010000000
100000100000000021000000010000
100000000010000021000010000000
020000100010000020000000000000
200000100010000002000000000000
000000100010000022000000000000
110000100010000011000000000000
000000100010000020000000020000
000000100010000020000020000000
200000100010000011000000000000
200000100010000010100000000000
200000100010000010010000000000
200000100010000010001000000000
200000100010000010000100000000
110000100010000020000000000000
101000100010000020000000000000
100100100010000020000000000000
100010100010000020000000000000
100001100010000020000000000000
200000010010000020000000000000
200000001010000020000000000000
200000000110000020000000000000
200000100001000020000000000000
200000100000100020000000000000
200000100000010020000000000000
110000010010000020000000000000
110000001010000020000000000000

110000000011000002000000000000
110000100001000020000000000000
110000100000100020000000000000
110000100000010020000000000000
011000100010000020000000000000
010100100010000020000000000000
010010100010000020000000000000
010001100010000020000000000000
000000210010000020000000000000
000000201010000020000000000000
000000200110000020000000000000
000000100021000020000000000000
000000100020100020000000000000
000000100020010020000000000000
100000000010000021000001000000
100000000010000021000000100000
100000100000000021000000001000
100000100000000021000000000100
100000101010000010000010000000
100000100110000010000010000000
100000100010100010000000010000
100000100010010010000000010000
211000100010000000000000000000
210100100010000000000000000000
210010100010000000000000000000
210001100010000000000000000000
200000010010000011000000000000
200000001010000011000000000000
200000000110000011000000000000
200000100001000011000000000000
200000100000100011000000000000
200000100000010011000000000000
210000000010000010000001000000
210000000010000010000000100000
210000100000000010000000001000
210000100000000010000000000100
201000000010000010000010000000
200100000010000010000010000000
200010000010000010000010000000
200001000010000010000010000000
201000100000000010000000010000
200100100000000010000000010000
200010100000000010000000010000
200001100000000010000000010000
200000201010000000000000000000
200000200110000000000000000000
200000100020100000000000000000
200000100020010000000000000000
101000100010000011000000000000
100100100010000011000000000000
100010100010000011000000000000
100001100010000011000000000000

110000100010000010100000000000
110000100010000010010000000000
110000100010000010001000000000
110000100010000010000100000000
100000100011000010000000010000
100000100010100010000000010000
100000100010010010000000010000
100000110010000010000010000000
100000101010000010000010000000
100000100110000010000010000000
000000100010000021100000000000
000000100010000021010000000000
000000100010000021001000000000
000000100010000021000100000000
000000100010000020000011000000
000000100010000020000010100000
000000100010000020000000011000
000000100010000020000000010100
200000100010000000000000011000
200000100010000000000000010100
200000100010000000000011000000
200000100010000000000010100000
200000000000000020000001010000
200000000000000020000000110000
200000000000000020000010001000
200000000000000020000010000100
200000100010000001100000000000
200000100010000001010000000000
200000100010000001001000000000
200000100010000001000100000000
220000000000000000000010010000
120000000010000010000010000000
120000100000000010000000010000
210000100000000001000000010000
210000000010000001000010000000
120000100000000001000000010000
100000000020001020000000000000
100000200000001020000000000000
200000100000001010000010000000
200000000010001010000000010000
100000000020000120000000000000
100000200000000120000000000000
200000100000000110000010000000
200000000010000110000000010000
100000100000000020000010000010
100000000010000020000000010010
100000100000000020000010000001
100000000010000020000000010001
120000000010000001000010000000
110000000000000011000010010000
020000100010000000000020000000
020000100010000000000000020000

0200001000100000020000000000000
1100000000000000200000100100000
0100002000100000100000100000000
0100001000200000100000000010000
1100001000100000000000200000000
1100001000100000000000000020000
1100000000200000000000100100000
1100002000000000000000100100000
2100001000000000000100000010000
2100000000100000001000100000000
1100002010100000000000000000000
1100001000201000000000000000000
2000000000000000101000100100000
1110000000100000100000100000000
1110001000000000100000000010000
2000000010100000000000200000000
2000001000001000000000000020000
2000001010000000000000100100000
2000000000101000000000100100000
1100001000100000011000000000000
0200001000100000011000000000000
2200000100100000000000000000000
2200001000010000000000000000000
1210001000100000000000000000000
2200001000001000000000000000000
2200000010100000000000000000000
1100001000210000000000000000000
1100002100100000000000000000000
2100000010000000100000000010000
2100000000000100010000010000000
1200000000100000010000001000000
1200001000000000010000000001000
0200002100100000000000000000000
0200001000210000000000000000000
2100000000100000000000000010010
2100001000000000000000010000010
1200002000000001000000000000000
1200000000200010000000000000000
1000000000200010000000000020000
1000002000000001000000020000000
0200000000000000200001001000000
0200000000000000100002000000100
0200000000000000100000000200100
1100000000000000200001001000000
1100000000000000100002000000100
1100000000000000100000000200100
1200000000000001002000000000000
1000000000000001000000020020000
1000000000000001002000020000000
1000000000000001002000000020000
0100000000000001000000020020000
1200000000000001000000020000000

010000000000001011000020000000

**C2 molecule NCMET(ND) WF**

20000002000200000200000000000000
22000002000200000000000000000000
20000002000000000200000000020000
20000000000200000200000020000000
21000002000100000100000000010000
21000001000200000100000010000000
20000001000100000200000010010000
20000002000200000000000000020000
20000002000200000000000020000000
22000002000000000200000000000000
22000000000200000200000000000000
10000001000200000210000010000000
10000002000100000210000000010000
00000002000200000220000000000000
02000002000200000200000000000000
11000002000200000110000000000000
20000000000200000220000000000000
20000002000000000220000000000000
00000002000200000200000020000000
00000002000200000200000000020000
20000000000200000200000000020000
20000002000000000200000020000000
20000002000200000020000000000000
11000002000110000200000000000000
11000001100200000200000000000000
01100002000200000200000000000000
20100001000200000100000010000000
20100002000100000100000000010000
21000002000100000100000000001000
21000001000200000100000001000000
10000002000100000210000000001000
10000001000200000210000001000000
20000002000110000110000000000000
20000001100200000110000000000000
21100002000200000000000000000000
11000001010200000200000000000000
11000002000101000200000000000000
10000002010200000100000010000000
10000002000201000100000000010000
21010002000200000000000000000000
10010002000200000110000000000000
20010001000200000100000010000000
20010002000100000100000000010000
21100002000000000200000000000000
21100000000200000200000000000000
20000002000200000000000011000000
20000002000200000000000000011000
11000002000200000101000000000000

10000002000210000010000000000010000
1000000210020000010000000100000000
2000000200000000002000000000011000
2000000000020000020000000110000000
0000000200020000002101000000000000
0000000200020000002000000000011000
0000000200020000002000000110000000
2000000101020000011000000000000000
2000000200010100011000000000000000
2000000200020000001100000000000000
2000000100010000020000000100010000
2000000100010000020000000010100000
2100100200020000000000000000000000
1000100200020000011000000000000000
2000100100020000010000000100000000
2000100200010000010000000000010000
0000000200020000002100100000000000
2100010200020000000000000000000000
1000010200020000011000000000000000
2000010100020000010000000100000000
2000010200010000010000000000010000
0000000200020000002100010000000000
2000000200010001010000000100000000
2000000100020001010000000000010000
1000000200010000020000000100000010
1000000100020000020000000000010010
2000000200020000000000000000010100
1100000200020000020000000000000000
1010000200020000020000000000000000
1001000200020000020000000000000000
1000100200020000020000000000000000
1000010200020000020000000000000000
2000000200020000011000000000000000
2000000200020000010100000000000000
2000000200020000010010000000000000
2000000200020000010001000000000000
2000000200020000010000100000000000
2000000110020000020000000000000000
2000000101020000020000000000000000
2000000200011000020000000000000000
2000000200010100020000000000000000
2000000100010000021000000000000010
2100000200010000010000000000000100
2100000100020000010000000001000000
1000000200010000021000000000000100
1000000100020000021000000001000000
2000000200020000000000000101000000
2000000200000000002000000000010100
2000000000020000020000000101000000
0000000200020000020000000000010100
0000000200020000020000000101000000
2000000100010000020000000100000100

20000001000100000200000000110000
22000002000000000000000000020000
22000000000200000000000020000000
22000001000100000000000010010000
20000000000000000200000020020000
21000001000000000100000010020000
21000000000100000100000020010000
12000001000200000010000010000000
12000002000100000010000000010000
02000002000200000020000000000000
20000000000200000000000020020000
20000002000000000000000020020000
11000001000100000110000010010000
22000000000200000000000000020000
22000002000000000000000020000000
22000000000000000200000020000000
22000000000000000200000000020000
02000002000200000000000020000000
02000002000200000000000000020000
10000001000000000210000010020000
10000000000100000210000020010000
22000000000200000020000000000000
22000002000000000020000000000000
00000002000000000220000000020000
00000000000200000220000020000000
22000001000100000000000010010000
11000002000000000110000000020000
11000000000200000110000020000000
00000001000100000220000010010000
01000002000100000120000000010000
01000001000200000120000010000000
00000002000200000000000020020000
20000000000000000220000020000000
20000000000000000220000000020000
10000001000200000010000010020000
10000002000100000010000020010000
00000002000200000020000000020000
00000002000200000020000020000000
21000000000100000120000000010000
21000001000000000120000010000000
01000002000100000100000020010000
01000001000200000100000010020000
02000000000200000220000000000000
02000002000000000220000000000000
12000000000100000210000000010000
12000001000000000210000010000000
00000002000000000200000020020000
00000000000200000200000020020000
22000000000000000220000000000000
20000001000100000020000010010000
20000000000200000020000000020000
20000002000000000020000020000000

00000002000000000220000020000000
00000000000200000220000000020000
02000000000200000200000000020000
02000002000000000200000020000000
02000001000100000200000010010000
11000000000200000110000000020000
11000002000000000110000020000000
20000002000000000200000000020000
20000000000200000200000020000000
02000002000000000200000000020000
02000000000200000200000020000000
12000002000100000100000000010000
12000001000200000100000010000000
21000002000100000010000000010000
21000001000200000010000010000000
11000001000100000200000010010000
11000002000200000000000000020000
11000002000200000000000020000000
02000002000200000110000000000000
11000002000200000020000000000000
20000001000100000110000010010000
22000002000000000110000000000000
22000000000200000110000000000000
11000002000000000200000000020000
11000000000200000200000020000000
10000001000200000100000010020000
10000002000100000100000020010000
01000002000100000210000000010000
01000001000200000210000010000000
10000002000100000120000000010000
10000001000200000120000010000000
21000000000100000210000000010000
21000001000000000210000010000000
20000002000000000110000000020000
20000000000200000110000020000000
00000002000200000110000000020000
00000002000200000110000020000000
20000000000200000110000000020000
20000002000000000110000020000000
11000000000200000220000000000000
11000002000000000220000000000000
11000000000200000200000000020000
11000002000000000200000020000000
21000002000100000001000000010000
21000001000200000001000010000000
22000002000110000000000000000000
22000001100200000000000000000000
20000000010100000200000010010000
20000001000001000200000010010000
12100002000200000000000000000000
12000001000000000100000010020000
12000000000100000100000020010000

10000001000000000120000010020000
10000000000100000120000020010000
21000001000000000010000010020000
21000000000100000010000020010000
02000002000000000110000000020000
02000000000200000110000020000000
11000002000000000020000000020000
11000000000200000020000020000000
02000001000100000110000010010000
11000001000100000020000010010000
20000000000000000110000020020000
01000000000100000210000020010000
01000001000000000210000010020000
22000000000000000110000000020000
22000000000000000110000020000000
11000000000000000200000020020000
11000000000200000000000020020000
11000002000000000000000020020000
01000002000100000010000020010000
01000001000200000010000010020000
00000000000200000110000020020000
00000002000000000110000020020000
02000000000200000110000000020000
02000002000000000110000020000000
11000000000000000220000000020000
11000000000000000220000020000000
12000000000100000120000000010000
12000001000000000120000010000000
22000000000000000000000020020000
20000000000000000020000020020000
00000000000000000220000020020000
02000000000000000200000020020000
12000001000000000010000010020000
12000000000100000010000020010000
02000002000000000020000000020000
02000000000200000020000020000000
02000001000100000020000010010000
11000000000000000110000020020000
02000002000000000000000020020000
02000000000200000000000020020000
22000000000000000020000000020000
22000000000000000020000020000000
01000000000100000120000020010000
01000001000000000120000010020000
00000000000200000020000020020000
00000002000000000020000020020000
02000000000200000020000000020000
02000002000000000020000020000000
02000000000000000220000000020000
02000000000000000220000020000000
02000000000000000110000020020000
11000000000000000020000020020000

0200000000000000000200000200020000

**N2 molecule NCMET(ND) WF**

2200000200020000020000000000000
2000000200020000020000000200000000
2000000200020000020000000000200000
2000000200020000022000000000000000
2200000000020000020000000200000000
2200000000020000020000000000200000
2200000000020000022000000000000000
2200000200000000020000000200000000
2200000200000000020000000000200000
2200000200000000022000000000000000
2200000200020000002000000000000000
2200000200020000000000000200000000
2200000200020000000000000000200000
0200000200020000022000000000000000
0200000200020000020000000200000000
0200000200020000020000000000200000
1100000200020000022000000000000000
1100000200020000020000000200000000
1100000200020000020000000000200000
2200000100010000020000000100100000
2100000200010000021000000000100000
2100000100020000021000000100000000
1200000100020000021000000100000000
1200000200010000021000000000100000
2200000200020000011000000000000000
2200000200020000010100000000000000
2200000200020000010010000000000000
1210000200020000020000000000000000
1201000200020000020000000000000000
1200100200020000020000000000000000
1200010200020000020000000000000000
2110000200020000020000000000000000
2101000200020000020000000000000000
2100100200020000020000000000000000
2100010200020000020000000000000000
2200000110020000020000000000000000
2200000101020000020000000000000000
2200000200011000020000000000000000
2200000200010100020000000000000000
2210000100020000010000000100000000
2201000100020000010000000100000000
2200100100020000010000000100000000
2200010100020000010000000100000000
2210000200010000010000000000100000
2201000200010000010000000000100000
2200100200010000010000000000100000
2200010200010000010000000000100000
1200000100020000020100000100000000

12000001000200000200100010000000
12000001000200000200010010000000
12000001000200000200001010000000
12000002000100000201000000010000
12000002000100000200100000010000
12000002000100000200010000010000
12000002000100000200001000010000
22000002000200000000000000011000
21000002000210000100000000010000
21000002100200000100000010000000
11000002000200000211000000000000
11000002000200000210100000000000
11000002000200000210010000000000
11000002000200000210001000000000
21100002000200000110000000000000
21010002000200000110000000000000
21001002000200000110000000000000
21000102000200000110000000000000
12000002000100000210000000001000
12000001000200000210000001000000
20000002000200000211000000000000
20000002000200000210100000000000
20000002000200000210010000000000
20000002000200000210001000000000
20000002000200000200000000011000
20000002000200000200000011000000
21000001000200000201000010000000
21000001000200000200100010000000
21000001000200000200010010000000
21000001000200000200001010000000
21000002000100000201000000010000
21000002000100000200100000010000
21000002000100000200010000010000
21000002000100000200001000010000
22000001010200000110000000000000
22000002000101000110000000000000
02000002000200000211000000000000
02000002000200000210100000000000
02000002000200000210010000000000
02000002000200000210001000000000
22000002000110000110000000000000
22000001100200000110000000000000
12000002010200000100000010000000
12000002000201000100000000010000
21000002010200000100000010000000
21000002000201000100000000010000
12000002100200000100000010000000
12000002000210000100000000010000
11000002000200000200000000011000
11000002000200000200000011000000
22000002000000000211000000000000
22000002000000000210100000000000

22000002000000000210010000000000
22000002000000000210001000000000
22000000000200000211000000000000
22000000000200000210100000000000
22000000000200000210010000000000
22000000000200000210001000000000
22000002000200000011000000000000
22000002000200000010100000000000
22000002000200000010010000000000
22000002000200000010001000000000
22000002000100010100000001000000
22000001000200010100000000010000
12000002000100000200000010000010
12000001000200000200000000010010
22000001000100000210000000000010
22000002000200000000000000010100
12000002000100000210000000000100
12000001000200000210000000100000
20000002000200000200000000010100
20000002000200000200000010100000
11000002000200000200000000010100
11000002000200000200000010100000
12000001000200000100000010020000
12000002000100000100000020010000
11000002000200000110000020000000
11000002000200000110000000020000
21000001000200000100000010020000
21000002000100000100000020010000
20000002000200000110000020000000
20000002000200000110000000020000
12000002000100000120000000010000
12000001000200000120000010000000
22000002000000000110000000020000
22000000000200000110000020000000
21000001000200000120000010000000
21000002000100000120000000010000
02000002000200000110000020000000
02000002000200000110000000020000
22000001000100000110000010010000
22000002000000000110000020000000
22000000000200000110000000020000
02000002000200000000000020020000
20000002000200000020000020000000
20000002000200000020000000020000
12000000000100000210000020010000
12000001000000000210000010020000
02000000000200000200000020020000
02000002000000000200000020020000
22000000000200000020000020000000
22000002000000000020000000020000
22000000000000000220000020000000
22000000000000000220000000020000

02000000000200000220000020000000
02000002000000000220000000020000
20000000000200000200000020020000
20000002000000000200000020020000
20000002000200000000000020020000
00000002000200000220000020000000
00000002000200000220000000020000
02000002000200000200000020000000
02000002000200000200000000020000
11000002000200000000000020020000
20000002000000000220000020000000
20000000000200000220000000020000
11000001000100000220000010010000
22000001000100000200000010010000
22000002000000000200000020000000
22000000000200000200000000020000
02000001000100000220000010010000
11000002000000000220000000020000
11000000000200000220000020000000
00000002000200000200000020020000
02000002000000000220000020000000
02000000000200000220000000020000
11000002000000000200000020020000
11000000000200000200000020020000
22000000000000000200000020020000
21000001000000000210000010020000
21000000000100000210000020010000
20000002000000000220000000020000
20000000000200000220000020000000
22000000000200000000000020020000
22000002000000000000000020020000
20000001000100000220000010010000
22000001000001000200000010010000
22000000010100000200000010010000
20000001100200000200000000020000
20000002000110000200000020000000
22100001000000000100000010020000
22100000000100000100000020010000
12000001000000000120000010020000
12000000000100000120000020010000
21000001000000000120000010020000
21000000000100000120000020010000
11000002000000000110000020020000
11000000000200000110000020020000
10000002000100000120000020010000
10000001000200000120000010020000
22000000000000000110000020020000
20000002000000000110000020020000
20000000000200000110000020020000
02000000000200000110000020020000
02000002000000000110000020020000
22000000000000000200000020020000

02000000000000000022000002002000
20000000000000000022000002002000
20000002000000000020000002002000
20000000000200000020000002002000
00000002000200000020000002002000
02000000000200000020000002002000
02000002000000000020000002002000
21100000000000000020000002002000
12100000000000000020000002002000
21010000000000000020000002002000
12010000000000000020000002002000
10000001000100010020000002002000
01000001000100010020000002002000
00200000000000000022000002002000
00000000200000000022000002002000
00000000000020000220000002002000
00000000000000002022000002002000
00000000000000000022000002002002
00000000000000000022000002202000
00000000000000000022000002002200
20000000000000000022000002002000
02000000000000000022000002002000

**O2 molecule NCMET(ND) WF**

220000200020000002000001001000
220000100010000002000000200200
200000200020000002200001001000
210000200010000002100001002000
210000100020000002100000200100
220000200011000002000001001000
220000110020000002000001001000
220000100021000002000000200000
220000210010000002000000002000
210000200021000002100001000000
210000210020000002100000001000
220000200020000000200001001000
220000000020000002000002101000
220000200000000002000001002100
220000200020000001100001001000
221000200010000001000001002000
220010200010000001000001002000
220001200010000001000001002000
220010100020000001000000200100
220001100020000001000000200100
210010200020000001100001001000
221000100020000001000000200100
210000200020100010000001002000
210000201020000001000000200100
211000200020000001100001001000
210100200020000001100001001000
020000200020000002200001001000
220000200000000002200001001000

22000000002000002200001001000
21000010002000002100001101000
21000020001000002100001001100
22000010001000002000001102000
22000010001000002000002001100
22000010102000001100001001000
22000020001010001100001001000
11000020002000002101001001000
11000020002000002110001001000
21000020102000002100000001000
21000020002010002100001000000
12000020001000002010001002000
12000010002000002010002001000
12000020001000002100001002000
12000010002000002100002001000
22010020001000001000001002000
22001020001000001000001002000
22000120001000001000001002000
22001010002000001000002001000
22000110002000001000002001000
22010010002000001000002001000
12100020002000002000001001000
22000020002000001010001001000
11000020002000002200001001000
12000020002100001000001002000
12000021002000001000002001000
22000020002000001001001001000
12000020001000002100001001100
12000010002000002100001101000
12000020002010002100001000000
12000020102000002100000001000
22000010102000002000001001000
22000020001010002000001001000
22000020002000001100001000100
22000020002000001100000101000
22000020101000002000000002000
22000010002010002000002000000
12010020002000002000001001000
22000020002000000101001001000
12000021002000002100000001000
12000020002100002100001000000
12010020002000001100001001000
20000020002000002110001001000
22000020002000002000000101000
22000020002000002000001000100
11000020002000002000001002100
11000020002000002000002101000
12000020002010001000001002000
12000020102000001000002001000
22000010002000102100001000000
22000020001000102100000001000
12000020001000002000002001001

1200001000200000020000010020010
2200001000100000021000010010010

22000010002000012100000100000000
22000020001000012100000000010000
12000020001000002000000200010001
12000010002000002000000100020001
22000010001000002100000100010001
21000120002000001100000100010000
21000020002100001000000100020000
21000021002000001000000200010000
22000011002000001100000100010000
22000020001100001100000100010000
21000021002000002100000000010000
21000020002100002100000100000000
22000011002000002000000100010000
22000020001100002000000100010000
22000020002000000110000100010000
12100020002000001100000100010000
12001020002000001100000100010000
12000120002000001100000100010000
21000020002001001000000100020000
21000020012000001000000200010000
22000010012000001100000100010000
22000020001001001100000100010000
21000020012000002100000000010000
21000020002001002100000100000000
12000020002001002100000100000000
12000020012000002100000000010000
22000010012000002000000100010000
22000020001001002000000100010000
22000020011000002000000000020000
22000010002001002000000200000000
12000020002001001000000100020000
12000020012000001000000200010000
10000020002000100200000200020000
10000020002000010200000200020000
01000020002000100200000200020000
01000020002000010200000200020000
00000020002000001200000200020010
00000020002000001200000200020001
01000020002000102000000200020000
01000020002000012000000200020000
10000020002000102000000200020000
10000020002000012000000200020000
21000000002000102000000200020000
21000000002000012000000200020000
12000000002000102000000200020000
12000000002000012000000200020000
21000020000000102000000200020000
21000020000000012000000200020000
12000020000000102000000200020000
12000020000000012000000200020000
20000020002000001000000200020010
20000020002000001000000200020001

020000200020000010000020020010

```
1201000200020000020000002002000
1200100200020000020000002002000
1200010200020000020000002002000
2110000200020000020000002002000
2101000200020000020000002002000
2100100200020000020000002002000
2100010200020000020000002002000
2200000200020000011000002002000
2200000200020000010100002002000
2200000200020000010010002002000
2200000200020000010001002002000
2200000200020000010000102002000
2200000110020000020000002002000
2200000101020000020000002002000
2200000200011000020000002002000
2200000100010000021000002002001
2200000200020000021000001001001
2200000100020001021000002001000
2200000200010001021000001002000
2200000200010100020000002002000
2200000200020000020000001102000
2200000200020000020000001012000
2200000200020000020000002001100
2200000200020000020000002001010
1200100200020000011000002002000
0200000200020000021001002002000
1200010200020000011000002002000
0200000200020000021000102002000
2100100200020000011000002002000
2000000200020000021001002002000
2100010200020000011000002002000
2000000200020000021000102002000
1200000200010000021000002002010
1200000100020000021000002012000
2100000200010000021000002002010
2100000100020000021000002012000
1100000101020000022000002002000
1100000200010100022000002002000
2000000200020000022000002001100
2000000200020000022000001102000
```

## C. INPUT EXAMPLE

```
***,Li2 NCMET(ND) wave function
gthresh,printci=0.00000001
print,orbitals,civector
r=2.6720 ang
geometry={li;li,li,r}
basis=def2-qzvp
hf
{mcscf
frozen,1,0,0,0,1,0,0,0
```

```
occ,7,4,4,2,7,3,3,2
wf,6,1,0
dont,orbital
select
con,200000000000000000000000000000
con,000000200000000000000000000000
con,000000000020000000000000000000
con,000000000000000020000000000000
con,020000000000000000000000000000
con,000000000000000002000000000000
con,000000000000000000000020000000
con,000000000000000000000000020000
con,110000000000000000000000000000
con,101000000000000000000000000000
con,100100000000000000000000000000
con,100010000000000000000000000000
con,100001000000000000000000000000
con,011000000000000000000000000000
con,010100000000000000000000000000
con,010010000000000000000000000000
con,010001000000000000000000000000
con,000000110000000000000000000000
con,000000101000000000000000000000
con,000000100100000000000000000000
con,000000000011000000000000000000
con,000000000010100000000000000000
con,000000000010010000000000000000
con,000000000000000011000000000000
con,000000000000000010100000000000
con,000000000000000010010000000000
con,000000000000000010001000000000
con,000000000000000010000100000000
con,000000000000000000000011000000
con,000000000000000000000010100000
con,000000000000000000000000011000
con,000000000000000000000000010100}
```
